# Membrane-based purification and recovery of phosphate and antibiotics by two-dimensional zeolitic nanoflakes†

**DOI:** 10.1039/d3ra02933f

**Published:** 2023-06-20

**Authors:** Tong Wu, Wenqian Chen, Minghong Wu, Yizhou Zhang

**Affiliations:** a Key Laboratory of Organic Compound Pollution Control Engineering, Ministry of Education, School of Environmental and Chemical Engineering, Shanghai University Shanghai 200444 China mhwu@shu.edu.cn; b Advanced Institute for Materials Research (WPI-AIMR), Tohoku University Sendai 980-8577 Japan zhang.yizhou.e4@tohoku.ac.jp

## Abstract

The pervasive presence of persistent contaminants in water resources, including phosphate and antibiotics, has attracted significant attention due to their potential adverse effects on ecosystems and human health. Adsorption membranes packed with metal–organic frameworks (MOFs) have been proposed as a potential solution to this challenge due to their high surface area to volume ratio, and the tailored functionality they can provide for selective purification. However, devising a straightforward method to enhance the stability of MOF membranes on polymer supports and manipulate their surface morphology remains challenging. In this study, we present a facile solution immersion technique to fabricate a ZIF-L adsorption membrane on commercial supports by leveraging the self-polymerization characteristics of dopamine. The simple coating methodology provides a polydopamine-lined interface that regulates the ZIF-L heteroepitaxial growth, along with tailored nanoflake morphology. Compared with crystals prepared in bulk solution, the sorbents grown on the membrane exhibit a higher saturation capacity of 248 mg g^−1^ of phosphate (∼80 mg phosphorus per g sorbent) and 196 mg g^−1^ for tetracycline in static adsorption experiments at 30 °C. Additionally, the membranes are capable of selectively removing 99.5% of the phosphate in simulant solutions comprising competitive background ions in various concentrations, and efficiently removing tetracycline. The result from the static adsorption experiments directly translates to a flow-through process, showcasing the utility of a composite membrane with a 3 μm thick active layer in practical adsorption applications. The facile solution immersion fabrication protocol introduced in this work may offer a more efficient paradigm to harness the potential of MOF composite membranes in selective adsorption and resource recovery applications.

## Abstract 2

This study presents a solution immersion method for fabricating zeolitic nanoflakes with controlled heteroepitaxial growth on membranes, enabling efficient phosphate and tetracycline uptake in high saturation capacities.

## Graphical abstract

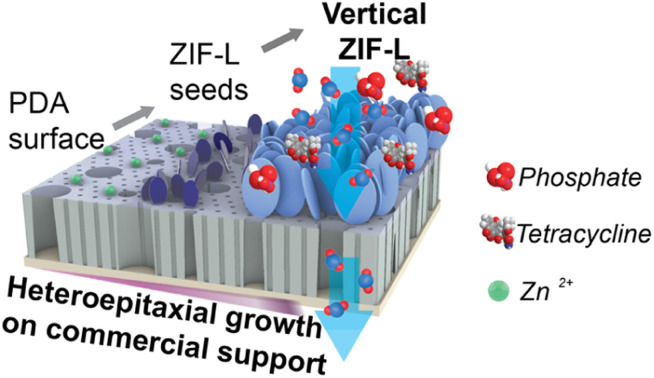

## 1. Introduction

The extensive growth of industrial and agricultural practices profoundly impacts freshwater supplies and disrupts the intricate balance of nutrient cycles.^1^ One detrimental outcome of these activities is the heightened presence of persistent and harmful contaminants in waterways.^2^ For instance, eutrophication, prompted by excessive nutrient enrichment of phosphorus leaching from agricultural land, leads to toxic blue-green algal blooms.^3,4^ Furthermore, the presence of antibiotics, such as tetracycline, complicates the purification by impeding the degradation of organic matter in domestic treatment systems.^5,6^ These challenges underscore the necessity to employ various techniques as an integral component of a more effective process tailored for purification purposes.^7–11^ Despite the ongoing management efforts, many conventional methods, including enhanced biological removal and chemical precipitation, struggle to meet the stringent regulations for micropollutants established by governmental agencies.^12^ For example, in the case of phosphate, the realistic output from conventional techniques is typically 2 to 3 orders of magnitude more concentrated than the suggested threshold (*i.e.*, 0.05 mg phosphorus per L).^13^ Further reduction in concentrations, however, is confined to a significant expenditure of chemical dopants for precipitation; alternatively, the enhanced biological process is expected to operate at narrow conditions unrealistic in practice.^14,15^ Consequently, a trade-off exists between purification efficacy and treatment cost.

Practically, wastewater treatment incurs considerable expenses, representing approximately 3% of global electricity consumption for household sewage discharge alone.^16^ Concurrently, alongside concerns about achieving a high level of removal, the focus on wastewater management is transitioning from removal to resource recovery, thereby allowing the cost of purification to be offset by the product.^1,17–19^ This emerging demand has initiated a paradigm shift towards more discriminatory purification characterized by single-species selectivity and minimal discharge. However, the gold standard for high-performance separation processes, such as membrane-based reverse osmosis or ion exchange, exhibits limited selectivity for solutes of similar size or charge characteristics.^20^ This limitation poses a challenge in nutrient and resource recycling when coexisting with other contaminants, such as heavy metals, which can adversely affect product value and have a negative impact on the environment quality and human health if inappropriately discharged.^21^

Adsorption leverages the affinity interaction between a functionalized surface and the targeted contaminants to achieve more selective separation.^4^ The introduction of a chemically selective ligand on the sorbent surface tailors the selectivity and the equilibrium binding constant, enabling the separation of desired solutes from a mixture of background molecules.^22^ Microporous resins, with diameters ranging from 0.2 to 3 mm, are commonly incorporated in adsorption-based packed beds due to their abundance of binding sites for prospective solute interactions.^23^ However, the extended diffusion pathway, often exceeding 100 μm, can compromise overall efficiency due to the prolonged mass transfer required for saturation.^24^ In this context, adsorptive membranes with pore sizes smaller than 1 μm offer a shorter diffusion distance,^25^ making them a promising solution for more efficient use of available binding sites, and achieving high-performance flow-through operations at increased throughputs. Current phosphate and tetracycline adsorption studies often involve mixed matrix membranes, where polymer materials are mixed with inorganic fillers. Graphitic and zeolitic materials are employed to create sorbents suitable for tetracycline, and in case of phosphate adsorption, transition metals such as lanthanum or zirconium and associated salts are directly incorporated into porous polymers.^26–29^ The use of metal sorbents for phosphate is because of the difficulty associated with achieving selective adsorption. Specifically, the hydration energy of the phosphate (−2773 kJ mol^−1^) is significantly higher than other competitive ions, *i.e.*, −347, −368, and −1090 kJ mol^−1^ of chloride, bicarbonate, and sulfate, respectively, where stronger binding with inner-sphere complexation with metal moieties becomes a necessary approach.^4,30^ Among these mixed matrix membranes, the porous structure is dedicated by the polymer framework, leading to potentially low binding density due to sparse sorbent distribution. Consequently, there is a need to develop adsorptive membranes with a controlled sorbent structure and distributions for more effective capturing and recycling of targeted solutes from water.^31^

Metal–organic frameworks (MOFs) are inorganic–organic hybrid materials characterized by their ordered porous structures with functionalized surface, rendering them highly suitable for various adsorption applications.^22,32–34^ Within the diverse library of MOF crystals, zeolitic imidazolate framework-L (ZIF-L) is noteworthy due to its cushion-shaped cavity packed within a 2D leaf-like crystal, as well as its abundant Zn–OH and charged imidazolate sites that are exposed to the solution. In particular, ZIF-L has a propensity for developing heteroepitaxial growth morphology on membranes, which packs densely distributed, vertically oriented, and water-insoluble crystals within the designated volume to enable separation with high permeance.^35,36^ Furthermore, compared to crystal developed in bulk solution, the growth on the confined surface enables the crystal miniaturization to endow a high surface area to volume ratio, providing a higher density of adsorption sites for a given membrane testbed volume.^37–39^

These advantageous characteristics for adsorption are demonstrated in recent studies highlighting defect-free ZIF-L adsorption membranes,^40–42^ based on substrates with specific surface chemistries to ensure the integrity and the crystal size of the MOF sorbent layer.^43–45^ For example, Xiao *et al.* proposed a ZIF-L infiltrated membrane by filling the ∼2 μm sized 2D-nanoflake crystals within porous silicon carbide ceramic membranes, to generate a hierarchically nanostructured hybrid matrix for efficient iodine removal.^25^ Similarly, Zhang and co-workers developed a self-crosslinking cellulose membrane that provides abundant hydroxyl moieties to facilitate the formation of a similarly sized ZIF-L percolated surface.^6^ While these studies have addressed sorbent morphology, the choice of substrate is limited from using commercial substrates, *i.e.*, polysulfone, cellulose acetate, and polyvinylidene fluoride (PVDF) membranes, however. Heterogeneous nucleation on commercial membranes lacking appropriate functional groups can lead to incomplete coverage during the initial growth stage, and contribute to the emergence of significant defects.^46^ To address this challenge, Chen and co-workers pioneered the use of APTES-functionalized TiO_2_ for the deposition of ZIF crystals on PVDF supports, employing a hydrothermal sol–gel coating protocol with prolonged heating procedures to achieve ultra-thin titania deposition on porous polymeric supports.^47^ However, the seed deposition method described in their study necessitates strict environmental control and kinetics requirements, limiting the applicability at larger scales.

In this study, we present a simple, room-temperature immersion technique for the preparation of oriented, ZIF-L adsorptive membranes on commercial polymer supports. To start with, polyvinylidene fluoride (PVDF) microfiltration membranes were immersed in an aqueous dopamine solution to enable self-polymerization, followed by drying under the ambient atmosphere. This creates an interface that regulates the *in situ* heteroepitaxial growth of the defect-free and structurally persistent ZIF layer. The high density of polydopamine anchoring sites and the unique adhesion characteristics of the phenolic-lined layer promote the formation of a Zn–OH lined, single-layer sorbent network.^48,49^ Vertically positioned ZIF-L in the current study possesses a crystal size that is smaller than bulk ZIF control, *i.e.*, 2 μm *vs.* 6 μm across the crystal surface along the *b*-direction, generating a surface area to volume ratio of 1.3 × 10^7^ m^2^ m^−3^, more than two times higher than the ZIF-L in bulk. Furthermore, the potential of ZIF-L depositions in parallel orientations to the film surface is systematically investigated and compared with the vertical crystals to locate the ideal fabrication conditions. The resulting adsorptive membrane frameworks effectively capture phosphate and tetracycline, as evidenced by static adsorptions in the presence of background ions and dynamic adsorption experiments. In particular, in static adsorption, the membranes achieve more than 99.5% removal of phosphate, and typically more than 90% of tetracycline adsorption in the presence of competitive ions. Further study reveals that MOF sorbents created using the polydopamine immersion methodology maintain their structure in pressure-driven operations, even after multiple adsorption and regeneration cycles.^50^ The facile immersion protocol demonstrated in this study offers a previously unexplored approach to harness the potential of MOF composite membranes in various purification and resource recovery applications.

## 2. Experimental

### 2.1 Materials

PVDF membranes with an averaged pore size of 0.22 μm were purchased from Tianjin Jinteng Experimental Equipment Co., Ltd. Dopamine hydrochloride (98%), tris(hydroxymethyl)aminomethane, potassium antimony tartrate (K_2_Sb_2_C_8_H_4_O_12_·3H_2_O, ≥99%) were purchased from Shanghai Macklin Biochemical Co., Ltd. Meanwhile, zinc nitrate hexahydrate (Zn(NO_3_)_2_·6H_2_O, 99%), 2-methylimidazole (Hmim, 98%), potassium dihydrogen phosphate (KH_2_PO_4_, ≥99%), sodium carbonate (Na_2_CO_3_, ≥98%), sodium sulfate (Na_2_SO_4_, ≥99%), sodium chloride (NaCl, ≥99.9%), ammonium tetramolybdate ((NH_4_)_6_Mo_7_O_24_·4H_2_O, 99%) were purchased from Shanghai Titan Scientific Co., Ltd. Hydrochloric acid (HCl, 36%), sodium nitrate (NaNO_3_, ≥99%) were purchased from Sinopharm Chemical Reagent Co., Ltd. Deionized water (DI, *R* = 18 MΩ cm) was used unless otherwise noted.

### 2.2 Fabrication of the vertical and parallel PVDF/PDA/ZIF-L membranes

PVDF membranes were rinsed in a bath of 50/50 (by volume) methanol–water solution for 30 min, followed by rinsing with excess DI water for 3 times. For a typical dopamine immersion solution, 0.25 g dopamine hydrochloride was dissolved in an aliquot of 100 mL Tris–HCl buffer (50 mM, pH = 8.5) to prepare a 2 g L^−1^ solution. The PVDF membranes were fully immersed in a dopamine solution housed within a beaker subject to magnetic stirring for 2 h. Following their removal from the dopamine solution, the dopamine-coated membranes underwent an air-drying process for 6 h, resulting in the acquisition of polydopamine-lined membranes, hereby referred to as PVDF/PDA. During drying, the environmental temperature was regulated between 21 to 24 °C, and the relative humidity was controlled from 50% to 80%. Membranes with vertically oriented ZIF-L (PVDF/PDA/ZIF-L) were prepared following a similar protocol reported in prior work.^6^ In particular, Zn(NO_3_)_2_·6H_2_O was blended with Hmim at different fractions (8 : 4, 8 : 2, 8 : 1, and 8 : 0.5) and dissolved in DI water. Subsequently, PVDF/PDA membranes were transferred to the precursor solution and let sit at room temperature for a predetermined amount of time. The PVDF/PDA/ZIF-L membranes were subsequently taken out and rinsed completely with DI water, followed by drying in a convection oven at 60 °C for 12 h and stored in a Petri dish for further use. The parallel PVDF/PDA/ZIF-L membranes were prepared by directly filtrating the ZIF-L bulk crystals, where the nanoflakes in bulk were prepared by dissolving 3.1 g Hmim and 1.4 g Zn(NO_3_)_2_·6H_2_O in aliquots of 75 mL water, respectively, followed by stir mixing for 1 h at room temperature. Following fabrication, bulk ZIF-L crystals were precipitated by centrifugation at 8000 rpm for 10 min, then rinsed with deionized water, and thereafter dried in a convective oven at 60 °C for 12 h. The bulk ZIF-L solution for filtration was prepared by diluting the solid crystals using an appropriate volume of water. After that, 2D crystal was deposited onto PVDF/PDA substrates by using vacuum filtration. Meanwhile, in the secondary growth study, the parallel ZIF-L seeding layer was allowed to develop a second layer on the existing crystal matrix. Developed ZIF-L membranes with crystals in parallel orientation were rinsed completely with DI water and were dried appropriately in a convection oven at 60 °C for 12 h.

### 2.3 Structural and chemical characterization

The surface morphology was characterized using a Zeiss Gemini field emission scanning electron microscope (SEM). Samples were prepared by trimming the air-dried membranes into 5 mm sized pieces, followed by mounting them on standard aluminum pin stubs and then sputter-coated with ∼4.0 nm of gold before loading them into the microscope chamber. Micrographs were usually captured at a working distance of 5.0 mm using an accelerating voltage of 1 kV. X-ray photoelectron spectroscopy (XPS) was used to conduct surface elemental analysis of the composite membrane, where the samples in smaller pieces, *e.g.*, 5 mm × 10 mm were loaded into a Thermo Scientific K-Alpha XPS chamber at high vacuum (2.0 × 10^−7^ mbar) for photoelectron spectrum collection. The elemental composition was analyzed using the numerical integration algorithm compiled in Advantage software package. Crystalline structures of the composite membrane were characterized using a Bruker D8 ADVANCE X-ray diffractometer at a scan rate of 10° min^−1^ in the range of 5 to 90° using Cu-α radiation. Additional membrane chemical characterization was performed by ATR-Fourier transform infrared spectroscopy (FT-IR, Thermo Scientific Nicolet iS5) in the range of 4000–400 cm^−1^. The concentration of phosphate and tetracycline were assessed using UV-vis spectrophotometry (SHIMADZU, UV-2600) assisted by ammonium molybdate, and the zeta potential was measured on an electro-kinetic analyzer (SurPASS, Anton Paar) by measuring the streaming potential values from pH 3 to 11, using 1 mM KCl solution as the background electrolyte, at room temperature. Furthermore, the ZIF-L crystal layer thickness, crystal size, permeable pore size distribution, and the effective adsorption area to volume ratio were estimated by using the Image-J software package. In order to acquire an accurate estimation, the ZIF-L layer thickness and the corresponding crystal size were measured from the average of different locations. ZIF-L layer pore size distribution was obtained by counting the area of spacing between crystals for at least 100 different points. Similarly, the ZIF-L layer effective adsorption area was acquired by measuring the number and corresponding dimensions of crystal in a pre-defined surface area (*i.e.*, 100 μm^2^).

### 2.4 Adsorption experiments

In the static adsorption experiments, pieces of membranes were exposed to phosphate and tetracycline solutions with a packing ratio of 0.5 g ZIF-L membrane per liter of solution. During the assessment experiment of the adsorption kinetics, membranes were immersed in 100 mL of phosphate and tetracycline solutions at various concentrations, namely 61, 123, and 184 mg L^−1^ for phosphate (corresponding to 20, 40, and 60 mg L^−1^ for total phosphorus) and 50, 100, and 200 mg L^−1^ for tetracycline, at a controlled environmental temperature of 30 °C. 2 mL of the sample was extracted for analysis during regular time intervals. Meanwhile, the experiment measuring equilibrium binding capacity as a function of crystal growth time involved immersing membranes in 10 mL aliquots of solutions with 184 ppm phosphate (∼60 mg L^−1^ phosphorus) and 200 mg L^−1^ tetracycline, carried out at a controlled temperature of 30 °C for 24 h. Similarly, in the binding isotherm experiment, membranes were immersed in 10 mL of solutions with different concentrations from 5 to 1500 mg L^−1^ for phosphate and tetracycline at controlled temperatures ranging from 30 to 50 °C, for a total duration of 12 h. These concentrations were deliberately selected to ensure that the isotherm study covers the range of retentate concentrations below the saturation point. Meanwhile, PVDF/PDA membranes were selected as the control. In case when the impact of background ions on the adsorption efficiency is assessed, simulant solutions containing different concentrations of Cl^−^, NO_3_^−^, SO_4_^2−^, and CO_3_^2−^ were prepared as the stock. Before being analyzed using UV-vis spectra, solutions were filtered through a 0.45 μm syringe filter to remove undissolved particles. More details of the calculation are listed in ESI.†

The dynamic binding, or breakthrough experiments, was based on a 47 mm diameter membrane testbed within a Swin-Lok cell with an active layer thickness of ∼3 μm. Solutions containing 6 mg L^−1^ phosphate (∼2 mg L^−1^ phosphorus), or alternatively 10 mg L^−1^ tetracycline, were permeated through the membrane driven by a syringe pump at a flow rate of 0.1 mL min^−1^. The solute concentration within the permeate solutions was monitored at regular time intervals using UV-vis spectroscopy. Following the membrane adsorption processes, the ZIF-L crystal was regenerated using the modified procedure similar to previously reported protocols.^43,44^ Specifically, membranes were regenerated by immersing them in an ethanol solution for 1 h after their exposure to 15 ppm phosphate and 5 ppm tetracycline. Meanwhile, the 1 h long regeneration using ethanolic NaOH solution at different concentrations was assessed. The regenerated membranes were then dried in a convection oven at 60 °C prior to being used for subsequent adsorption experiments.

## 3. Results and discussion

### 3.1 Fabrication of oriented PVDF/PDA/ZIF-L membranes

**Fig. 1:**
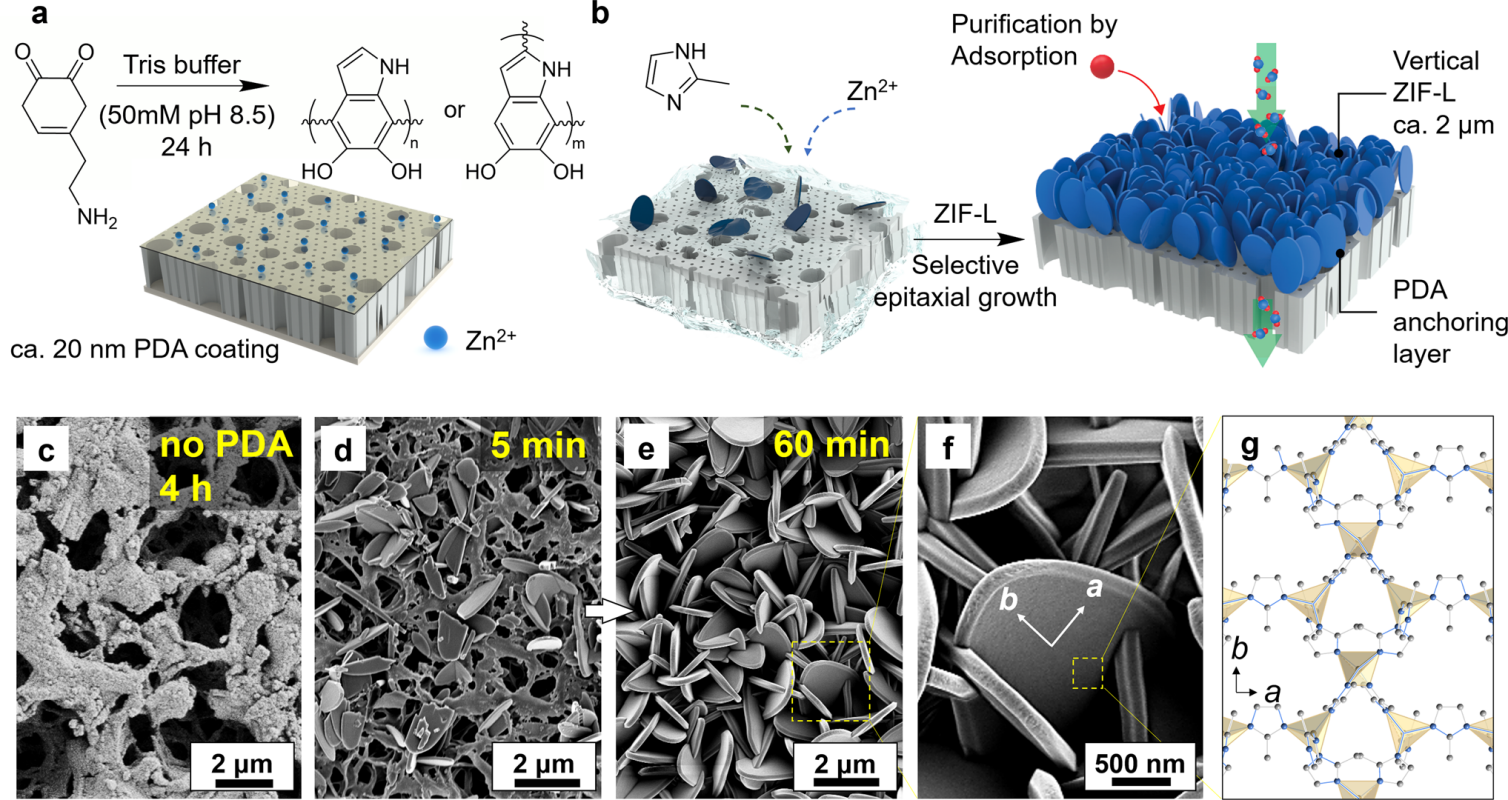
Schematic illustration of adsorption membrane with two-dimensional ZIF-L nanoflakes and micrographs of their surface structure. (a) Polydopamine-coated thin-film provides a high density of Zn^2+^ anchoring sites atop PVDF support. (b) By immersing the support in a solution of ZIF precursor, small-sized nanocrystals are allowed to grow within a confined surface, along with a heterogeneous epitaxial pattern due to rapid crystallization. The growth yields a self-supported, *ca.* 3 μm thick zeolitic matrix of the elliptic fan-like structure. (c) In cases where there is no surface anchoring, growth develops in a higher degree of freedom, which leads to the absence of densely arranged crystals. (d) Crystals developed as a function of time on a PDA functionalized PVDF membrane, where crystals are grown from small size seed crystals in 5 min to (e) vertical crystals that span across the entire surface area in 60 min, with (f) magnified micrograph for an individual ZIF-L crystal, which provides a high surface area of binding sites demonstrated in (g).

The regulation of crystal orientation is essential for attaining optimal membrane permeance and managing mass transfer in adsorption processes. In this study, the vertical alignment of ZIF-L crystals was initiated by modulating the crystal growth conditions, where a polydopamine (PDA) layer was introduced to establish chemical anchoring sites for subsequent heteroepitaxial growth as shown in Fig. 1.^51^ Here, the PDA coating was achieved using a facile immersion procedure at room temperature, allowing dopamine to self-polymerize in basic buffer solution. This procedure involved catechol oxidation into dopamine quinone, followed by intermolecular cyclization, oxidative oligomerization, and rearrangements, leading to the self-assembly of a persistent and thin polymer coating, as demonstrated in Fig. 1a.^48^ Subsequent characterization suggests that the PDA layer was uniformly deposited on the substrate (Fig. S1†), with the characteristic elements (N and O) visualized under the energy dispersive X-ray spectroscopy (EDS) displaying a comprehensive coverage atop the PVDF surface structure. Because the dopamine polymerization is confined to the surface, nanoscale morphology from the support is retained despite the chemical treatment and the polymer coating. Crystal growth was then conducted in the precursor solution at room temperature ranging from 21 to 24 °C. Fig. 1b depicts the schematic presentation of ZIF-L layer growth on the PDA-modified substrate after immersion in the precursor solution. The polyphenol and amino groups of the PDA layer provided a multitude of anchoring sites for ZIF-L growth by coordinating with zinc metal ions.^50^ This interaction contributed to a mechanically robust PVDF/PDA/ZIF-L membrane with vertical crystal orientation, which could be easily handled and proved valuable for subsequent adsorption and permeation experiments (Fig. S2†).

In instances where crystal growth occurred on the surface without functionalization, the growth direction is free to explore on the plane, resulting in the formation of large, randomly distributed leaf-like crystals (*i.e.*, >6 μm) shown in Fig. S3.† Distributed crystals on surface infer prolonged growth did not guarantee effective anchoring, and in fact, crystals were absent after 4 h growth, as demonstrated in Fig. 1c. Conversely, the presence of PDA on the surface contributed the emergence of seed crystals well attached within 5 min of growth (Fig. 1d).^52^ Prior studies suggest that ZIF-L crystals exhibit accelerated growth kinetics in the *b*-out-of-plane direction with suitable surface nucleation, as the rapid development on membrane among surface normal inhibits the expansion in other directions.^53,54^ This phenomenon is supported by the transition to a dense pack of crystals after 60 min growth, where the ZIF-L displayed an anisotropic arrangement along the long-axis, as demonstrated in Fig. 1e. The magnified image is listed in Fig. 1f. In this orientation, the surface along *b*-direction is fully exposed during the adsorption to ensure efficient mass transfer and high surface area for solutes to interact with (Fig. 1g).

**Fig. 2:**
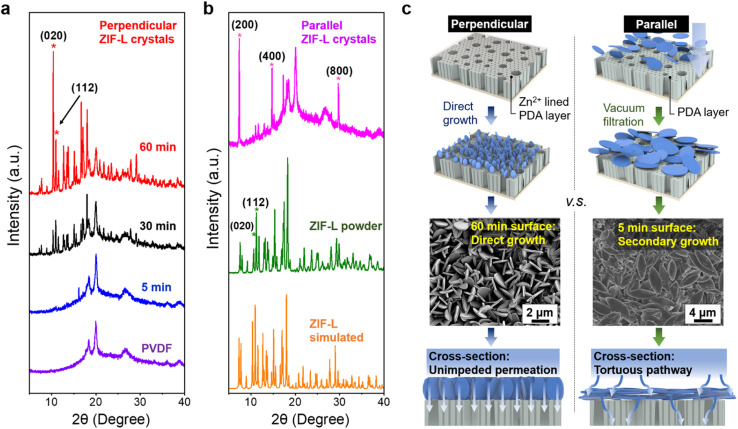
(a) XRD indicates the alignment of perpendicular ZIF-L crystals after prolonged growth. (b) The XRD spectra of ZIF-L in different states of alignments, presented in parallel orientation and in isotropic bulk powder. (c) Schematic illustration shows the growth process of perpendicular ZIF-L and the targeted surface evolution over time, in comparison with the preparation of parallel crystals. For membranes with parallel ZIF-L orientation, crystals are attached to the support by straightforward vacuum filtration on PDA functionalized support. Here, distinct permeation pathways have been generated due to the variations in crystal orientations.

A more detailed analysis of crystal orientation was performed using X-ray diffraction spectroscopy (XRD). Notably, the XRD peaks of the vertical ZIF-L membrane aligned with those of simulated ZIF-L diffraction patterns, and ZIF powder in bulk, shown in Fig. 2, indicating the successful synthesis of ZIF-L on the PVDF substrate. Notably, the occurrence of perpendicular crystal orientations is signified by the evolution of (020) peak over the growth time. The intensity of several XRD peaks deviated from those of simulated and powder ZIF-L, providing evidence of the variation in the degree of orientation. In particular, the orientation can be quantified using the crystallographic preferred orientation (CPO) index, as described in the ESI,† with the (020) and (112) reflections selected for the calculation as described in prior studies.^54^ A CPO value of 1 indicates the existence of a more preferred direction. In this case, the calculated CPO of 1.5 suggests the formation of out-of-plane vertical crystals, which is considerably higher than the value of 0.39 observed for the ZIF powder control.

We further investigated ZIF-L crystals in parallel orientation. By employing a simple vacuum filtration method, parallel ZIF-L layers were produced on PVDF/PDA substrates, with PDA serving as the surface primer for physical attachment, as illustrated in Fig. 2c. It should be noted that in comparison with the channels generated by perpendicular-oriented crystals, this morphology generates a tortuous permeation pathway for the prospective flow-through experiment. Additionally, when dealing with membranes with parallel crystals, the membranes become notably fragile, resulting in large cracks during the processing. Regardless, XRD analysis provides valuable information corroborating the observed nanostructures and their consistent parallel orientation, as evidenced by the presence of (*h*00) peaks, where *h* is a multiplier of 2, depicted in Fig. 2b. Interestingly, during secondary growth, miniaturized ZIF-L crystals emerge as perpendicular structures, yielding less pronounced signals in parallel orientation within the diffraction spectrum, shown in Fig. S4.† Although membranes with parallel crystals are not employed in subsequent flow-through experiments due to their poor mechanical property and tortuous permeation mechanism, the observation of secondary structures may provide guidance for sorbent design in the preparation of miniaturized crystals.

### 3.2 Tailored sorbent surface morphology

**Fig. 3:**
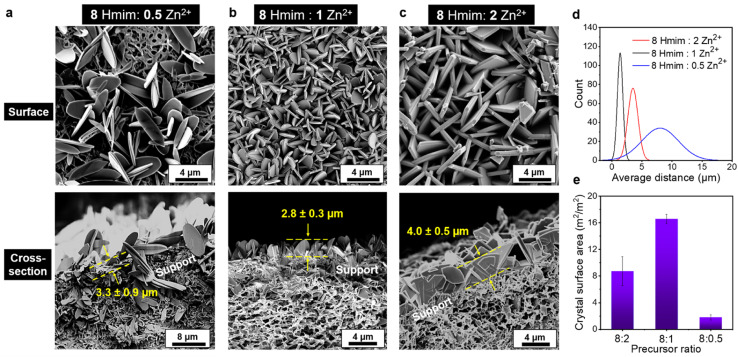
SEM micrographs demonstrate the structural evolution of the PVDF/PDA/ZIF-L membrane as a function of the Zn^2+^ concentration, while maintaining consistent growth time and Hmim precursor concentration. (a) The growth at an 8 : 0.5 (Hmim : Zn^2+^) molar ratio generates a surface network with low crystal packing density, leaving a significant portion of the support membrane surface area uncovered. (b) Increase in Zn^2+^ concentration corresponds to smaller crystals with improved surface area available for adsorption. Here, uniformly deposited, vertically oriented crystals are densely packed atop the support. (c) Further increase in Zn^2+^ undergoes a morphological change, which is characterized by edge coarsening and more sparsely distributed crystals. (d) Among different precursor ratios examined, ZIF-L layer prepared from the 8 : 1 (Hmim : Zn^2+^) molar ratio offers the shortest diffusion distance required for a solute to travel within the membrane before encountering another active site on the sorbent. (e) The same 8 : 1 formula offers the greatest sorbent surface area per unit of membrane surface area.

The context of the present work demonstrates the potential of preparing ZIF-L composite membranes with crystals in both parallel and vertical orientations. Intriguingly, the morphology of the vertical ZIF-L membrane can be facilely controlled by adjusting the Zn^2+^ concentration and growth time, which is crucial for the development of ZIF-L crystals in the desired morphology, and maximizing the sorbent site density on the substrates. As demonstrated in Fig. 3a, a lower Zn^2+^ ratio (8 Hmim : 0.5 Zn^2+^) has resulted in larger crystals. The uncovered surface area of the support membrane was substantial, potentially due to the limited cation sites available that impeded the heterogeneous nucleation during the initial growth stage. In turn, the calculated adsorption surface area (2 m^2^ m^−2^ membrane surface area) was lower than that observed for other molar ratios. By increasing the Zn^2+^ concentration, more reactive sites became available, promoting the formation of a dense and smaller-sized ZIF-L layer, as shown in Fig. 3b. The projected distance between crystals subsequently decreased, and its distribution became more uniform, improving the adsorption surface area ratio to 17 m^2^ m^−2^ of the membrane. Further increasing cation concentration may induce rapid crystallization that outweighs the impact of nucleation sites, resulting in crystals with significant size distributions with a larger distance between crystals (Fig. 3c), and a lower adsorption area ratio of 9 m^2^ m^−2^ of the membrane. Detailed statistics of the averaged distance distribution between each crystal and the calculated surface area are shown in Fig. 3d and e, respectively.

The kinetics of crystal growth is an intricate process comprising several steps, from rate-limiting nucleation to crystal growth, each governed by various influencing factors that shape the overall mechanism.^55^ Previous research shows that crystal formation begins with temporary, metastable complexes before moving onto the creation of stable and ordered crystal structures advantageous for high-capacity adsorption.^56^ However, extended growth of ZIF-L (*i.e.*, >2 h) correlates with complete surface coverage, driving the generation of a denser, semi-permeable ZIF-L membrane.^52^ In this exploration of ZIF-L heteroepitaxial growth with a constant ratio of 8 : 1 (Hmim : Zn^2+^) on the polydopamine surface, a non-monotonic rise in adsorption capacity over time is identified with aggregating crystals, as shown in Fig. S5.† While high binding capacity is preferred, crystal aggregation from the surface with longer growth time may disrupt flow systems by causing excessive pressure drops. In particular, vertical ZIF-L membranes with a growth duration of 60 min maintain structural integrity under pressure-driven flow, making them suitable for absorptive applications with a high hydraulic permeance of ∼430 L m^−2^ h^−1^ bar^−1^ (Fig. S6†). This information provides crucial guidelines for fabricating the ZIF-L sorbent membrane for subsequent static and dynamic adsorption experiments.

### 3.3 Chemical characterization

**Fig. 4:**
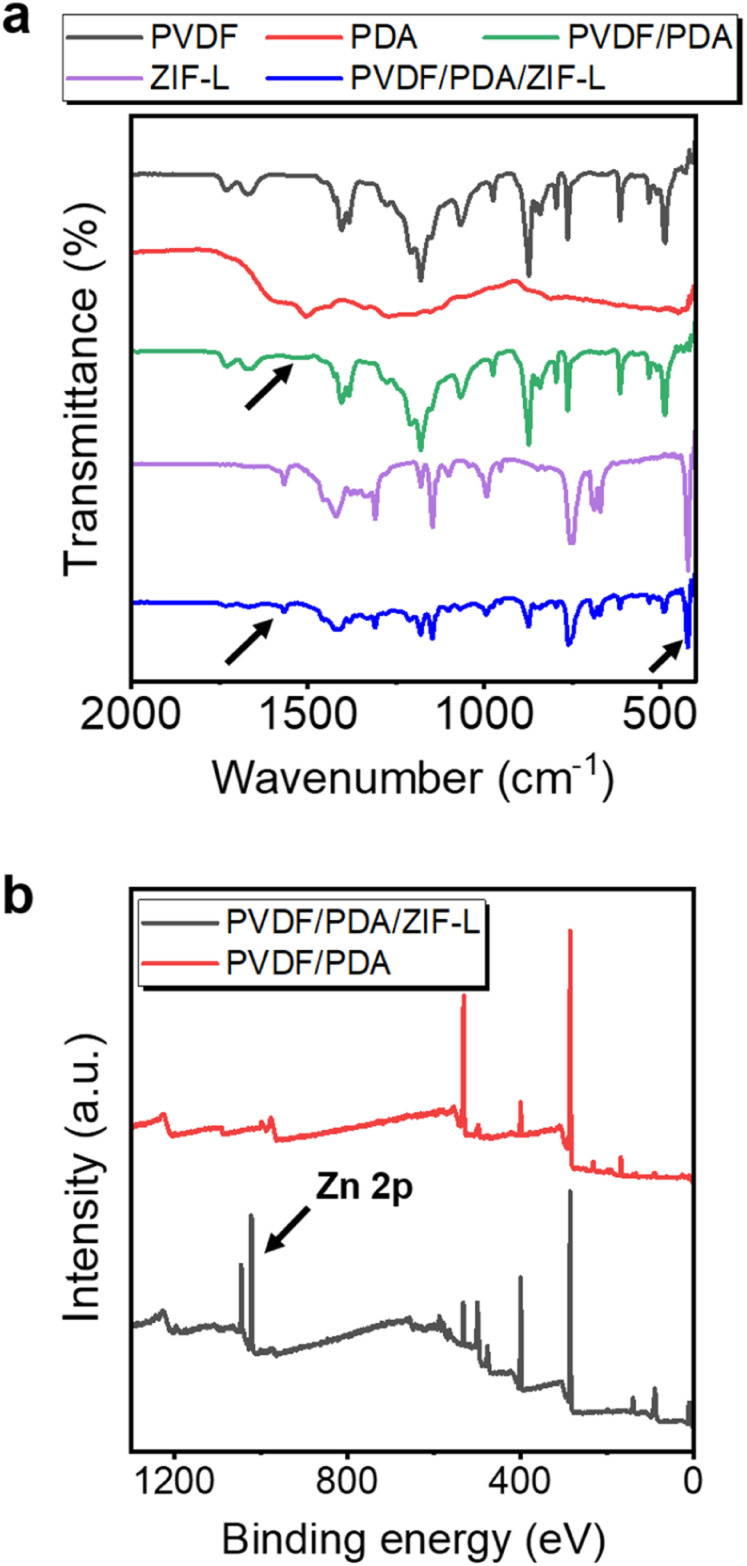
Chemical analysis for the ZIF-L composite membrane. (a) The FTIR monitors the surface functionalization and reveals the emergence of an amine peak at 1510 cm^−1^, indicating the coating of the PDA layer. Peaks at ∼1570 and 421 cm^−1^ are consistent with the presence of imidazole and Zn^2+^ within the ZIF-L framework. (b) An XPS survey spectra depicting the enhanced photoelectron intensity of Zn^2+^ on the membrane, informing the successful anchoring of binding sites on the surface.

The adhesion of PDA and ZIF-L layers to the substrate was further evidenced by chemical characterizations. To this end, attenuated total reflectance Fourier transform infrared spectroscopy (ATR-FTIR) was employed to analyze various functional groups on the sorbent and intermediate layers, as shown in Fig. 4a. In comparison to the PVDF membrane, several new transmission signals were observed on the PVDF/PDA membrane after modification. The peak at ∼1510 cm^−1^ is attributed to the N–H bond of the PDA.^48^ Similarly, the PVDF/PDA/ZIF-L membrane displayed new peaks at 420 and 1570 cm^−1^, attributed to the Zn–N and the C=N bond, respectively. This confirmed the presence of a coordinate bond between Zn^2+^ and the ligand, as well as the imidazole ring.^57^ Additionally, variation in the chemistry of the PVDF/PDA and ZIF-L coated membranes was determined by using X-ray photoelectron spectroscopy (XPS) analysis, as shown in Fig. 4b. The enhanced photoelectron intensity in Zn 2p from 1020 to 1040 eV suggested the presence of Zn^2+^ upon the heteroepitaxial growth, after prolonged soaking in DI water to rinse off residual ions.

### 3.4 Adsorption kinetics and adsorption isotherms

**Fig. 5:**
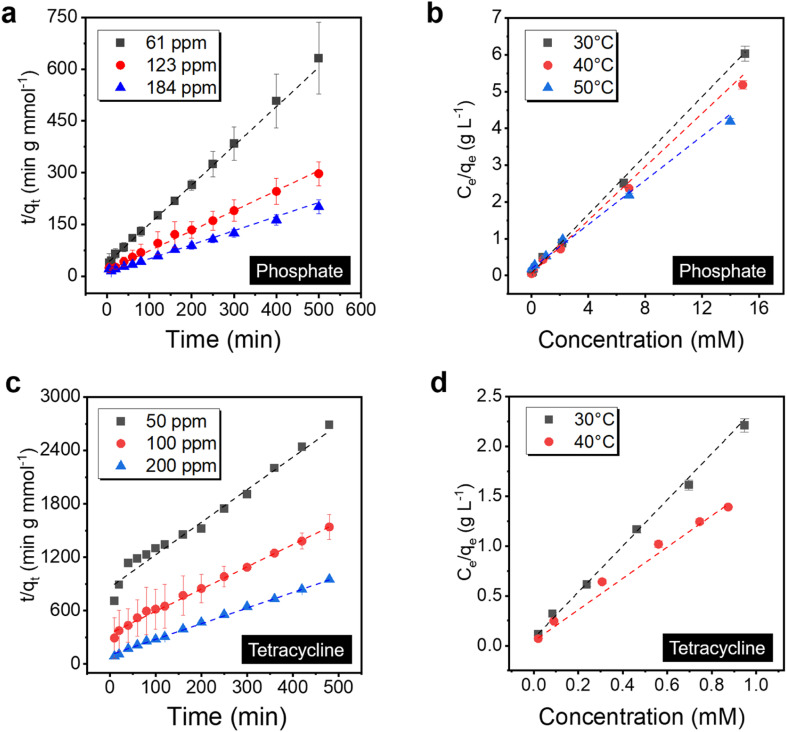
Adsorption isotherm and kinetics study of (a) and (b) phosphate and (c) and (d) tetracycline for the PVDF/PDA/ZIF-L membrane at various temperatures. The kinetics in the static adsorption experiment follows a pseudo-second order model, as indicated by the dashed line for both (a) phosphate and (c) tetracycline. The equilibrium capacities were determined as a function of the solute concentrations, and the dashed lines present the fitting using Langmuir isotherm. From the fitting, the inverse value of the slope suggests a saturation capacity of 2.6 mmol g^−1^ and 0.44 mmol g^−1^ for (b) phosphate and (d) tetracycline, respectively.

Adsorption kinetics and isotherm analysis are essential to elucidate the property of the sorbent systems, which guides critical parameters to optimize the process and a better understanding of the materials parameter governing the performance. To start with, the binding behavior of the PVDF/PDA/ZIF-L membrane towards phosphate was investigated by fitting with various adsorption kinetics models (*i.e.*, pseudo-first order and pseudo-second order) as well as adsorption isotherms (Langmuir and Freundlich isotherms).^58–61^ The membranes were immersed in solutions and placed on a shaker to minimize the external mass transfer resistance during the static adsorption experiment. In particular, within the 3 μm thick ZIF-L layer, the phosphate adsorption kinetics is better fitted by the pseudo-second order model (*R*^2^ = 99.9%), indicating that the adsorption kinetics is limited by chemical reactions, as illustrated in Fig. 5a.^62^ Additional kinetics analysis is provided in Fig. S7 and Table S1.† The result suggests the ligand exchange may control the overall adsorption kinetics.

Meanwhile, the saturation capacity is determined by the number of available sites, and the ZIF-L sorbent may saturate once the binding surface area is fully occupied. To validate this point, phosphate adsorption isotherms were further fitted by the Langmuir and Freundlich isotherms at different adsorption temperatures. As displayed in Fig. 5b, the Langmuir isotherm model (*R*^2^ = 99.9%) exhibits a better correlation than the Freundlich isotherm (*R*^2^ = 89.5%, shown in Fig. S8†), confirming the monolayer adsorption, with a maximum binding capacity 2.6 mmol g^−1^ (∼80 mg phosphorus per g) at 30 °C and a thermodynamically favored binding with the equilibrium constant 6.73 L mmol^−1^. Meanwhile, PVDF/PDA possesses no significant adsorption, as shown in Fig. S9.† Additional data are listed in Table S2.† Furthermore, when the adsorption temperature increased from 30 to 50 °C, the calculated maximum adsorption capacities of the PVDF/PDA/ZIF-L membrane increased to 3.7 mmol g^−1^ (115 mg phosphorus per g) at a price of the decreased equilibrium constant, confirming the exothermic adsorption behavior.^64^ This high capacity outweighs typical inorganic sorbents, such as zinc-aluminum layered double hydroxides with a saturation capacity of 0.61 mmol g^−1^,^63^ as well as similar ZIFs, with details listed in Table S3.† The achievement of a higher surface area by preparing ∼2 μm sized crystals by heteroepitaxial growth instead of ∼8 μm sized crystals in bulk (Fig. S10†) may have contributed to such a higher capacity.

XPS spectra were analyzed to further investigate the phosphate adsorption mechanism. As illustrated in Fig. S11,† a new P 2p peak emerged at ∼133.2 eV after phosphate adsorption on the PVDF/PDA/ZIF-L membrane. The occurrence of the P 2p peak suggests the presence of chemical bonding between phosphate and the ZIF-L.^65^ Furthermore, the high-resolution Zn 2p spectrum was analyzed to verify the existence of chemical bonding. Prior to the phosphate adsorption experiment, Zn 2p_3/2_ and Zn 2p_1/2_ orbitals exhibited two peaks at 1021.9 eV and 1044.9 eV, respectively. These two Zn 2p peaks shifted towards higher energy by 0.3 eV after adsorption, which is attributed to the formation of inner-sphere complexation due to strong interactions between Zn^2+^ to HPO_4_^2−^ and H_2_PO_4_^−^ ions.^66^ In turn, coupled with the adsorption kinetics analysis, although synergistic mechanisms, for example, hydrogen bonding and electrostatic interactions may contribute a part in the phosphate adsorption on ZIF-L, the inner-sphere complexation dominated by ligand exchange is likely governing the overall ZIF-L adsorption.^43^

The uptake behavior of the PVDF/PDA/ZIF-L membrane towards tetracycline was investigated, revealing adsorption kinetics akin to that of phosphate. As shown in Fig. 5c, the tetracycline adsorption kinetics were better fitted by the pseudo-second order model, which had higher correlation coefficients than the pseudo-first order, listed in Fig. S7.† Detailed data is provided in Table S4.† The tetracycline adsorption isotherms were subsequently fitted using the Langmuir and Freundlich isotherm models at 30 °C and 40 °C, respectively, with corresponding data listed in Table S5.† As displayed in Fig. 5d, the Langmuir isotherm model (*R*^2^ = 99.8%) fits better than the Freundlich isotherm model, as shown in Fig. S8.† The results indicated a preference for monolayer adsorption over multilayer adsorption, although the π–π interactions play a role in facilitating the surface–solute interaction.^44^ As the adsorption temperature increased from 30 to 40 °C, the calculated maximum tetracycline capacities of ZIF-L increased from 0.44 to 0.66 mmol g^−1^, outperforming similar inorganic sorbents as listed in Table S6.† Further, the associated XPS spectra revealed shifts in N=C and O=C peaks towards higher energy by 0.3 eV and 0.4 eV, respectively, after adsorption, as shown in Fig. S12.† These shifts may be attributed to the hydrogen bonding connecting between tetracycline and the PVDF/PDA/ZIF-L membrane coupled with the π–π interactions.^67^ Consequently, the synergistic effects enable efficient capture of tetracycline and phosphate from water. Further investigation simulating real-world conditions, such as those found in wastewater, is undertaken in the background ion adsorption experiment discussed in the following section.

### 3.5 Effect of background ions

**Fig. 6:**
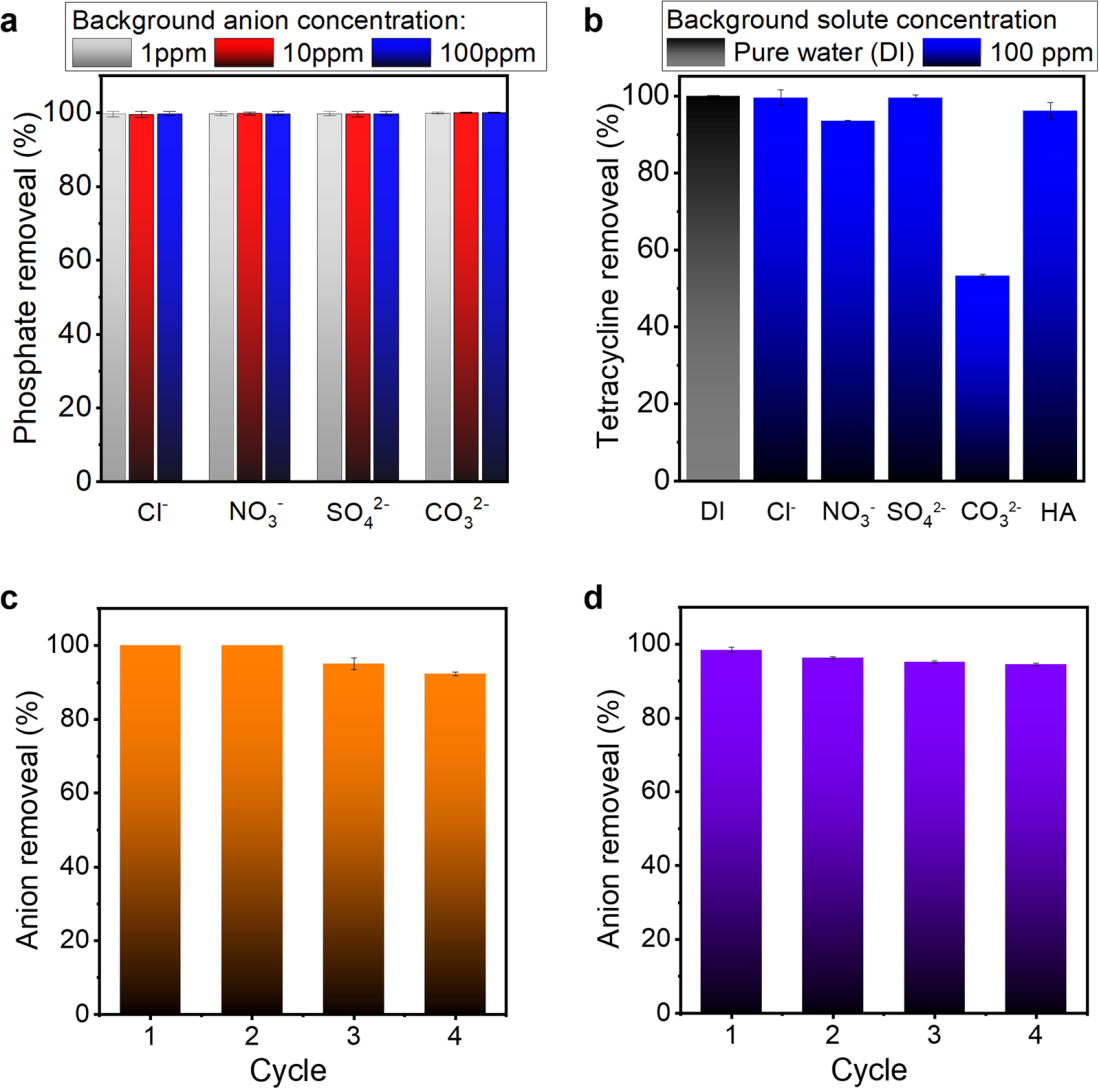
A survey of anion removal efficiency of the PVDF/PDA/ZIF-L membranes. The static anion uptake was determined by placing the membrane in an aliquot of 15 ppm phosphate and 5 ppm tetracycline solutions, respectively, with a packing ratio of 0.5 g of ZIF-L per L of solution. (a) The phosphate removal experiment was conducted with various background anions in different concentrations from 1 to 100 ppm, while (b) the tetracycline adsorption experiment was performed in a range of competitive anions but with a consistent concentration of 100 ppm. The membranes were repeatedly immersed in excess ethanol for regeneration. Following the first adsorption, the regeneration cycle was repeated at least 4 times for (c) phosphate and (d) tetracycline.

Wastewater water is often complex in composition, comprising various background ions and organic compounds that can negatively interfere with the contaminant removal. Therefore, it is vital to investigate the influence of other ions or organic compounds on adsorption in practical applications. For phosphate adsorption, typical anions (Cl^−^, NO_3_^−^, SO_4_^2−^ and CO_3_^2−^) with various concentrations were added to the solution to examine their effects on the adsorption process. As shown in Fig. 6a, by increasing concentrations of background anions from 1 to 100 mg L^−1^, the PVDF/PDA/ZIF-L membrane retained a nearly 100% phosphate removal efficiency. This excellent performance and high selectivity can be attributed to the strong affinity between active Zn^2+^ sites and phosphate, resulting from robust chemical bonding of inner sphere complexation, and a high p*K*_sp_ value 32 of Zn_3_(PO_4_)_2_.^4^ For tetracycline, the adsorption relies on hydrogen bonding, electrostatic and aromatic–aromatic interactions, making the efficiency susceptible to certain background molecules. We examined the impact of different ions (Cl^−^, NO_3_^−^, SO_4_^2−^ and CO_3_^2−^) and solutes such as humic acid (HA) on the corresponding adsorption behavior. As illustrated in Fig. 6b, Cl^−^, NO_3_^−^, and SO_4_^2−^ had minor impacts on the tetracycline adsorption process, whereas the presence of carbonate (CO_3_^2−^) strongly decreased the overall efficiency. This degeneration can be attributed to two factors. First, the addition of carbonate increased the solution pH to ∼10, which enhanced the electrostatic repulsion between the negatively charged composite membrane and the tetracycline (as detailed in Fig. S13†). Second, the bicarbonate anion is a hydrogen bond acceptor as well as a donor, therefore can disrupt the hydrogen bonding between the tetracycline and ZIF-L. Nonetheless, overall, effective removal of tetracycline was achieved. This performance, coupled with the high hydraulic permeance of composite membranes, demonstrates its potential for applications such as dynamic adsorption in real water environments.

### 3.6 Regeneration of PVDF/PDA/ZIF-L membrane

Regeneration behavior is another crucial factor in evaluating the practical applicability of the adsorption membranes. Phosphate and tetracycline adsorption–regeneration experiments were conducted, as depicted in Fig. 6c and d. PVDF/PDA/ZIF-L membranes were immersed in ethanol solution for regenerating the adsorption sites after their exposure to 15 ppm phosphate and 5 ppm tetracycline.^44^ The observed removal efficiencies are moderately consistent, with less than 8% loss after 4 consecutive regeneration cycles, indicating that the interaction between solute and membrane could be effectively disrupted under mild conditions and regenerate the membrane for further adsorption. The loss in capacity may be attributed to a reduction in the number of active adsorption sites, for example, the Zn-phosphate chemical bonding is stable enough from complete dissociation under the given condition. However, the partial desquamation of ZIF-L under more intense alkaline stimuli for regeneration, such as 0.01 M NaOH, may initiate an irreversible reduction in the number of adsorption sites due to the structural damage, shown in Fig. S14 and S15,† enacted by caustic chemical on both the polymer support and the ZIF-L crystal.

### 3.7 Dynamic breakthrough curves

**Fig. 7:**
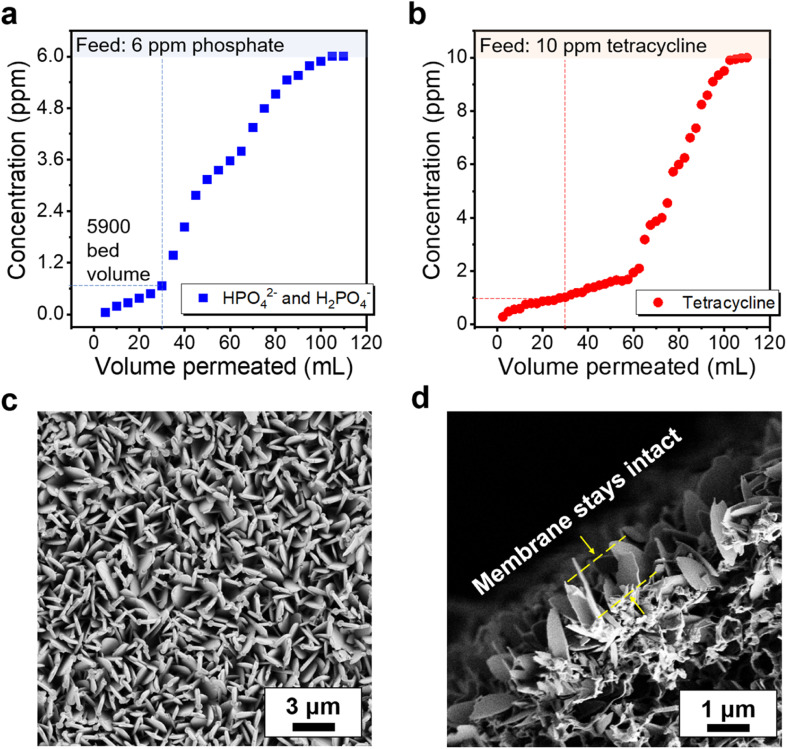
Dynamic breakthrough curves show anion removal as a function of permeated volume at a solution pH of 7.5. The membranes were placed in a syringe holder with feed solution aliquot containing (a) 6 ppm phosphate and (b) 10 ppm tetracycline facing the ZIF-L surface layer, at a packing ratio of 70 mg ZIF per L solution. The sample was permeated at a consistent flux of ∼0.9 L m^−2^ h^−1^ and was collected periodically as the permeation progressed. The dashed line represents the feed concentration where saturation is observed after ∼120 mL of solution permeated through the membranes. The surface (c) and cross-section (d) of the ZIF-L membrane maintain high integrity after the dynamic adsorption experiment.

Detailed isotherms and kinetics studies have identified that the use of ZIF-L crystals has demonstrated high saturation capacity with unimpeded performance in solutions with background ions. These findings highlight the need for further exploration of dynamic adsorption characteristics, as they aim to enable continuous purification better suited for myriad applications. As the result demonstrated from the breakthrough experiment in Fig. 7, ZIF-L composite membranes perform well in capturing phosphate and tetracycline. By using a ZIF-L membrane with only 3 μm active layer thickness, at the initial stage, the phosphate and tetracycline removal rates reach 99% and 98%, respectively, which is due to the high adsorption surface area provided by the vertically orientated ZIF-L crystals on the membrane. The removal rate decreases as adsorption sites become more occupied on the membrane and reach 90% after 30 mL solution is permeated, equivalent to 5900 bed volume. To better understand the adsorption characteristics and mechanisms in the dynamic adsorption process, a model that couples the Langmuir isotherm and second-order adsorption kinetics was introduced to estimate the maximum adsorption capacity and the dynamic adsorption rate constant.^68^ By assuming no axial dispersion, the breakthrough behavior can be predicted (Fig. S16†). The dynamic adsorption rate constants for phosphates and tetracycline are calculated as 1.3 × 10^−3^ and 5.4 × 10^−4^ L mg^−1^ min^−1^, and the adsorbent maximum adsorption capacity have achieved 16.8 mg g^−1^ for phosphate and 35.2 mg g^−1^ for tetracycline, respectively. The higher phosphate dynamic adsorption rate constant leads to faster occupancy of vacancies, which can explain the differences in the breakthrough curves. Notably, the ZIF-L composite membranes maintain high integrity after the dynamic adsorption experiment, as shown in Fig. 7c and d, confirming the robust anchoring and exceptional adhesion properties of the PDA attached ZIF-L layer in flow-through conditions.

## 4. Conclusions

In conclusion, our work presents a combined polymer coating and nanocrystal growth fabrication strategy for developing phosphate and antibiotic adsorptive membranes. In particular, ZIF-L crystals were anchored and oriented with perpendicular morphology on support in a straightforward manner. Optimized membrane characteristics useful for adsorption were identified by introducing PDA as a prime layer and depositing crystals in different methodologies. Specifically, critical information from the structural analysis revealed that small ZIF-L nanoflakes grow following a heteroepitaxial pattern and are packed within a 3 μm thick sorbent layer using a facile solution immersion paradigm. The membranes subsequently provide efficient adsorption in simulant solutions, achieving more than 99.5% removal of phosphate and efficient tetracycline removal, including in the presence of background electrolytes. Moreover, the binding is primarily reversible with gentle stimuli for regeneration. The results also suggest that fabricating ZIF-L crystals leads to a higher saturation capacity compared to bulk-solution crystals, and the subsequent flow-through experiment demonstrates the potential of using these ultra-thin sorbent membranes in practical applications. Further research is needed to examine the process parameters for these membranes, such as their propensity for fouling and antibacterial performance. The facile ZIF-L composite membrane fabrication developed in this work holds promise for manufacturing groundwater and wastewater remediation devices, particularly in cases where high-throughput, solute-selective adsorption is required.

## Author contributions

T. Wu, W. Chen, M. Wu and Y. Zhang conceived the ideas and designed the research. T. Wu conducted the experiments. T. Wu and Y. Zhang contributed to the manuscript writing.

## Conflicts of interest

The authors declare that they have no competing interests.

## Supplementary Material

RA-013-D3RA02933F-s001

## References

[cit1] Mayer B. K., Baker L. A., Boyer T. H., Drechsel P., Gifford M., Hanjra M. A., Parameswaran P., Stoltzfus J., Westerhoff P., Rittmann B. E. (2016). Environ. Sci. Technol..

[cit2] Schwarzenbach R. P., Escher B. I., Fenner K., Hofstetter T. B., Johnson C. A., von Gunten U., Wehrli B. (2006). Science.

[cit3] Conley D. J., Paerl H. W., Howarth R. W., Boesch D. F., Seitzinger S. P., Havens K. E., Lancelot C., Likens G. E. (2009). Science.

[cit4] Wu B., Wan J., Zhang Y., Pan B., Lo I. M. C. (2020). Environ. Sci. Technol..

[cit5] Wang Y., Kuntke P., Saakes M., van der Weijden R. D., Buisman C. J. N., Lei Y. (2022). Water Res..

[cit6] Li Z., Gou M., Yue X., Tian Q., Yang D., Qiu F., Zhang T. (2021). J. Hazard. Mater..

[cit7] Wang J., Zhang G., Qiao S., Zhou J. (2023). Sci. Total Environ..

[cit8] Sen A., Bakshi B. R. (2023). Sci. Total Environ..

[cit9] Sendrowski A., Boyer T. H. (2013). Desalination.

[cit10] Cao Y., Li X., Zhang L. (2023). Int. J. Electrochem. Sci..

[cit11] Xie M., Shon H. K., Gray S. R., Elimelech M. (2016). Water Res..

[cit12] Trebuch L. M., Sohier J., Altenburg S., Oyserman B. O., Pronk M., Janssen M., Vet L. E. M., Wijffels R. H., Fernandes T. V. (2023). Water Res..

[cit13] Phosphorus, Agriculture & the Environment, https://www.pubs.ext.vt.edu/424/424-029/424-029.html, accessed June 2023

[cit14] Mulkerrins D., Dobson A., Colleran E. (2004). Environ. Int..

[cit15] Cao X., Harris W. (2008). Environ. Sci. Technol..

[cit16] Zhang X., Liu Y. (2022). Chem. Eng. J..

[cit17] Larsen T. A., Hoffmann S., Lüthi C., Truffer B., Maurer M. (2016). Science.

[cit18] Li W.-W., Yu H.-Q., Rittmann B. E. (2015). Nature.

[cit19] Zodrow K. R., Li Q., Buono R. M., Chen W., Daigger G., Dueñas-Osorio L., Elimelech M., Huang X., Jiang G., Kim J.-H., Logan B. E., Sedlak D. L., Westerhoff P., Alvarez P. J. J. (2017). Environ. Sci. Technol..

[cit20] Elimelech M., Phillip W. A. (2011). Science.

[cit21] Geise G. M., Paul D. R., Freeman B. D. (2014). Prog. Polym. Sci..

[cit22] Burtch N. C., Jasuja H., Walton K. S. (2014). Chem. Rev..

[cit23] Gustavsson P. E., Larsson P. O. (1999). J. Chromatogr. A.

[cit24] Weidman J. L., Mulvenna R. A., Boudouris B. W., Phillip W. A. (2015). Langmuir.

[cit25] Xiao H., Zhou H., Feng S., Gore D. B., Zhong Z., Xing W. (2021). J. Membr. Sci..

[cit26] Chen L., Liu F., Wu Y., Zhao L., Li Y., Zhang X., Qian J. (2018). Chem. Eng. J..

[cit27] Xia W.-J., Guo L.-X., Yu L.-Q., Zhang Q., Xiong J.-R., Zhu X.-Y., Wang X.-C., Huang B.-C., Jin R.-C. (2021). Chem. Eng. J..

[cit28] Biswas B. K., Inoue K., Ghimire K. N., Ohta S., Harada H., Ohto K., Kawakita H. (2007). J. Colloid Interface Sci..

[cit29] Koilraj P., Sasaki K. (2017). Chem. Eng. J..

[cit30] Custelcean R., Moyer B. A. (2007). Eur. J. Inorg. Chem..

[cit31] Kubota N., Konno Y., Miura S., Saito K., Sugita K., Watanabe K., Sugo T. (1996). Biotechnol. Prog..

[cit32] Boyd P. G., Chidambaram A., García-Díez E., Ireland C. P., Daff T. D., Bounds R., Gładysiak A., Schouwink P., Moosavi S. M., Maroto-Valer M. M., Reimer J. A., Navarro J. A. R., Woo T. K., Garcia S., Stylianou K. C., Smit B. (2019). Nature.

[cit33] Liu J., Thallapally P. K., McGrail B. P., Brown D. R., Liu J. (2012). Chem. Soc. Rev..

[cit34] Canivet J., Fateeva A., Guo Y., Coasne B., Farrusseng D. (2014). Chem. Soc. Rev..

[cit35] Jeong Y., Hong S., Jang E., Kim E., Baik H., Choi N., Yip A. C., Choi J. (2019). Angew. Chem., Int. Ed..

[cit36] Kwon H. T., Jeong H.-K., Lee A. S., An H. S., Lee J. S. (2015). J. Am. Chem. Soc..

[cit37] Tanaka S., Fujita K., Miyake Y., Miyamoto M., Hasegawa Y., Makino T., Van der Perre S., Cousin Saint Remi J., Van Assche T., Baron G. V. (2015). J. Phys. Chem. C.

[cit38] Chocarro-Ruiz B., Pérez-Carvajal J., Avci C., Calvo-Lozano O., Alonso M. I., Maspoch D., Lechuga L. M. (2018). J. Mater. Chem. A.

[cit39] Xiao X., Zou L., Pang H., Xu Q. (2020). Chem. Soc. Rev..

[cit40] Wang S., Liu J., Pulido B., Li Y., Mahalingam D., Nunes S. P. (2020). ACS Appl. Nano Mater..

[cit41] Yang K., Hu S., Ban Y., Zhou Y., Cao N., Zhao M., Xiao Y., Li W., Yang W. (2021). Sci. Bull..

[cit42] Zhu J., Li H., Hou J., Liu J., Zhang Y., Van der Bruggen B. (2020). AIChE J..

[cit43] Huang C., Zhang H., Zheng K., Zhang Z., Jiang Q., Li J. (2021). Sci. Total Environ..

[cit44] Peng H., Xiong W., Yang Z., Cao J., Jia M., Xiang Y., Hu Q., Xu Z. (2021). Chem. Eng. J..

[cit45] Koo W.-T., Jang J.-S., Qiao S., Hwang W., Jha G., Penner R. M., Kim I.-D. (2018). ACS Appl. Mater. Interfaces.

[cit46] Brown A. J., Brunelli N. A., Eum K., Rashidi F., Johnson J. R., Koros W. J., Jones C. W., Nair S. (2014). Science.

[cit47] Hou J., Sutrisna P. D., Zhang Y., Chen V. (2016). Angew. Chem., Int. Ed..

[cit48] Jiang J., Zhu L., Zhu L., Zhu B., Xu Y. (2011). Langmuir.

[cit49] Lee H., Scherer N. F., Messersmith P. B. (2006). Proc. Natl. Acad. Sci. U. S. A..

[cit50] Wang X.-p., Hou J., Chen F.-s., Meng X.-m. (2020). Sep. Purif. Technol..

[cit51] Wu X., Zhou C., Dong C., Shen C., Shuai B., Li C., Li Y., An Q., Xu X., Mai L. (2022). Nano Res..

[cit52] Li H., Hou J., Bennett T. D., Liu J., Zhang Y. (2019). J. Mater. Chem. A.

[cit53] Li Y. S., Bux H., Feldhoff A., Li G. L., Yang W. S., Caro J. (2010). Adv. Mater..

[cit54] Zhang X., Li H., Miao W., Shen Q., Wang J., Peng D., Liu J., Zhang Y. (2019). AIChE J..

[cit55] Stock N., Biswas S. (2012). Chem. Rev..

[cit56] Stavitski E., Goesten M., Juan-Alcañiz J., Martinez-Joaristi A., Serra-Crespo P., Petukhov A. V., Gascon J., Kapteijn F. (2011). Angew. Chem., Int. Ed..

[cit57] Low Z.-X., Yao J., Liu Q., He M., Wang Z., Suresh A. K., Bellare J., Wang H. (2014). Cryst. Growth Des..

[cit58] Langmuir I. (1918). J. Am. Chem. Soc..

[cit59] Ho Y. S., McKay G. (1999). Process Biochem..

[cit60] Weber W. J., Morris J. C. (1963). J. Sanit. Eng. Div., Am. Soc. Civ. Eng..

[cit61] Yoon S.-Y., Lee C.-G., Park J.-A., Kim J.-H., Kim S.-B., Lee S.-H., Choi J.-W. (2014). Chem. Eng. J..

[cit62] Putra E. K., Pranowo R., Sunarso J., Indraswati N., Ismadji S. (2009). Water Res..

[cit63] Cheng X., Huang X., Wang X., Zhao B., Chen A., Sun D. (2009). J. Hazard. Mater..

[cit64] Thagira Banu H., Karthikeyan P., Meenakshi S. (2018). Int. J. Biol. Macromol..

[cit65] Dake L., Baer D., Friedrich D. (1989). J. Vac. Sci. Technol., A.

[cit66] Gupta N. K., Saifuddin M., Kim S., Kim K. S. (2020). J. Mol. Liq..

[cit67] Cui J., Xu X., Yang L., Chen C., Qian J., Chen X., Sun D. (2020). Chem. Eng. J..

[cit68] Xu Z., Cai J.-g., Pan B.-c. (2013). J. Zhejiang Univ., Sci., A.

