# Supplementary material for: Membrane-based purification and recovery of phosphate and antibiotics by two-dimensional zeolitic nanoflakes
Source: RSC Adv. 2023 Jun 20;13(27):18799–811. doi: 10.1039/d3ra02933f (PMC10281495; doi:10.1039/d3ra02933f)
Supplement: RA-013-D3RA02933F-s001 [file RA-013-D3RA02933F-s001.pdf]

Electronic Supplementary Material (ESI) for RSC Advances.
This journal is © The Royal Society of Chemistry 2023

Supplementary Information for

Membrane-based Purification and Recovery of Phosphate and Antibiotics by Two-dimensional Zeolitic Nanoflakes

Tong Wu,[a] Wenqian Chen,[a] Minghong Wu,*[a] and Yizhou Zhang*[a,b]

[a]Key Laboratory of Organic Compound Pollution Control Engineering, Ministry of Education, and School of Environmental and Chemical Engineering, Shanghai University, Shanghai 200444, China.

[b]Advanced Institute for Materials Research (WPI-AIMR), Tohoku University, Sendai, 980-8577, Japan

* Corresponding authors.

Email: mhwu@shu.edu.cn (M. Wu)
Email: zhang.yizhou.e4@tohoku.ac.jp (Y. Zhang)

1. Supplementary Equations **S2**

1.1 Adsorption capacity and removal rate calculation S2

1.2 Adsorption kinetics model S3

1.3 Adsorption isotherm model S3

1.4 Crystallographic preferred orientation (CPO) index S4

1.5 Coupling the Langmuir isotherm and second-order adsorption kinetics S4

2. Supplementary Figures **S5**

Figure S1 Surface morphology and elemental analysis S5

Figure S2 The photograph and micrograph demonstrating robbustness S5

Figure S3 SEM micrograph demonstrates a ZIF-L crystal S6

Figure S4 The in-plane alignment of parallel ZIF-L crystals S6

Figure S5 Adsorption capacity of the ZIF-L membranes over growth time S7

Figure S6 Pure water flux of perpendicular oriented PVDF/PDA/ZIF-L S7

Figure S7 Fittings of the pseudo-first order adsorption model S8

Figure S8 Fittings of the Freundlich isotherm model S8

Figure S9 Solute concentration in the solution before and after adsorption S9

Figure S10 Illustration for the in-plane dimension and the thickness of ZIF-L....S9

Figure S11 XPS analysis before and after phosphate adsorption S10

Figure S12 XPS analysis before and after tetracycline adsorption S10

Figure S13 Relative abundance of charged species and Zeta potential S11

Figure S14 The phosphate removal efficiency after regeneration S11

Figure S15 Surface micrograph of the membrane after being regenerated S12

Figure S16 Application of model on dynamic adsorption S12

3. Supplementary Tables S13

Table S1 Calculated kinetic parameters of phosphate adsorption S13

Table S2 Calculated isotherm parameters of phosphate adsorption S13

Table S3 Phosphate adsorption of sorbents recorded in literature S14

Table S4 Calculated kinetic parameters of tetracycline adsorption S14

Table S5 Calculated isotherms parameters of tetracycline adsorption S15

Table S6 Tetracycline adsorption of other adsorbents recorded in literature S15

References S16

1. Supplementary Equations

1.1 Adsorption capacity and removal rate calculation

The equilibrium capacity, denoted as q_e (mg g^{-1}) is computed using:

$$q_e = \frac{(C_0 - C_e)V}{m} \qquad (Eq. S1)$$

In this equation, C_0 (mg L^{-1}) is the initial solute concentration while C_e (mg L^{-1}) is the concentration at equilibrium. Similarly, the adsorption capacity q_t at given time t (mg g^{-1}) is calculated as:

$$q_t = \frac{(C_0 - C_t)V}{m} \qquad (Eq.S2)$$

Here, C_t (mg L^{-1}) is the retentate concentration at t (min), V (L) represents the solution volume, and m refers to the sorbent mass denoted with unit (g). Further, the removal rate, R (%), can be determined by the following equation:

$$R = \frac{C_0 - C_t}{C_0} \times 100\% \qquad (Eq.S3)$$

1.2 Calculations related with adsorption kinetics model

Adsorption kinetic modeling using the Pseudo-first order rate law is given as:

$$\ln\left(q_e - q_t\right) = \ln q_e - K_1 t \qquad (Eq.S4)$$

Where q_e (mg g^{-1}) is the equilibrium adsorption capacity, and q_t (mg g^{-1}) is the adsorption capacity at time t. K_1 (min^{-1}) denotes the pseudo first order rate constant. Alternatively, analysis based on the pseudo-second order model is expressed as:

$$\frac{t}{q_t} - \frac{t}{q_e} = \frac{1}{K_2 {q_e}^2} \qquad (Eq.S5)$$

Where K_2 (min^{-1}) is the pseudo-second order rate constant. For intraparticle diffusion, the model is derived from the linearization of the curve $q_t = f(t^{0.5})$, which is expressed as by the formula:

$$q_t = K_i t^{\frac{1}{2}} + C \qquad (Eq.S6)$$

Where K_i (mg g^{-1} $min^{-0.5}$) is the intraparticle rate constant.

1.3 Adsorption isotherm model

The Langmuir isotherm model is employed to calculate the equilibrium capacity as a function of solute concentration, and it is given as:

$$q_e = \frac{Q_m K_L C_e}{1 + K_L C_e} \qquad (Eq.S7)$$

In this model, Q_m (mg g^{-1}) is Langmuir adsorption capacity and K_L (L mg^{-1}) is Langmuir equilibrium constant. Alternatively, Freundlich adsorption isotherm is mathematically shown as:

$$q_e = K_F C_e^{\frac{1}{n}} \qquad (Eq. S8)$$

In this context, K_F (L mg^{-1}) is the Freundlich adsorption capacity, and n^{-1} indicates the adsorption intensity.

1.4 Crystallographic preferred orientation (CPO) index

The degree of orientation can be calculated by using the crystallographic preferred orientation (CPO) index. Specifically, the (020) and (112) reflections were chosen for the calculation,[1] which the formula can be articulated as follows:

$$CPO_{020/112} = \frac{\left[\left(\frac{I_{020}}{I_{112}}\right)_M - \left(\frac{I_{020}}{I_{112}}\right)_S\right]}{\left(\frac{I_{020}}{I_{112}}\right)_S} \qquad (Eq. S9)$$

In this equation, the I stands for the intensity of the corresponding reflection at the selected peak. The subscript M refers to the ZIF-L membrane, and S represents the simulation of the ZIF-L powder.

1.5 Coupling the Langmuir isotherm and second-order adsorption kinetics

A coupled equation, also known as the Thomas model, has been introduced to describe the adsorption performance in the dynamic process. This approach is implemented due to the satisfactory fit of the phosphate and tetracycline adsorption characteristics by the pseudo-second order adsorption kinetics and the Langmuir adsorption isotherm model. Note that this model assumes there is no axial dispersion. The formula is expressed as follows:

$$\ln\left(\frac{C_0}{C_t} - 1\right) = \frac{k_T}{F}(Q_m m - C_0 t) \qquad (Eq. S10)$$

Here, F is the inlet flow rate (L min^{-1}) and K_T (L mg^{-1} min^{-1}) is the dynamic rate constant.

2. Supplementary Figures

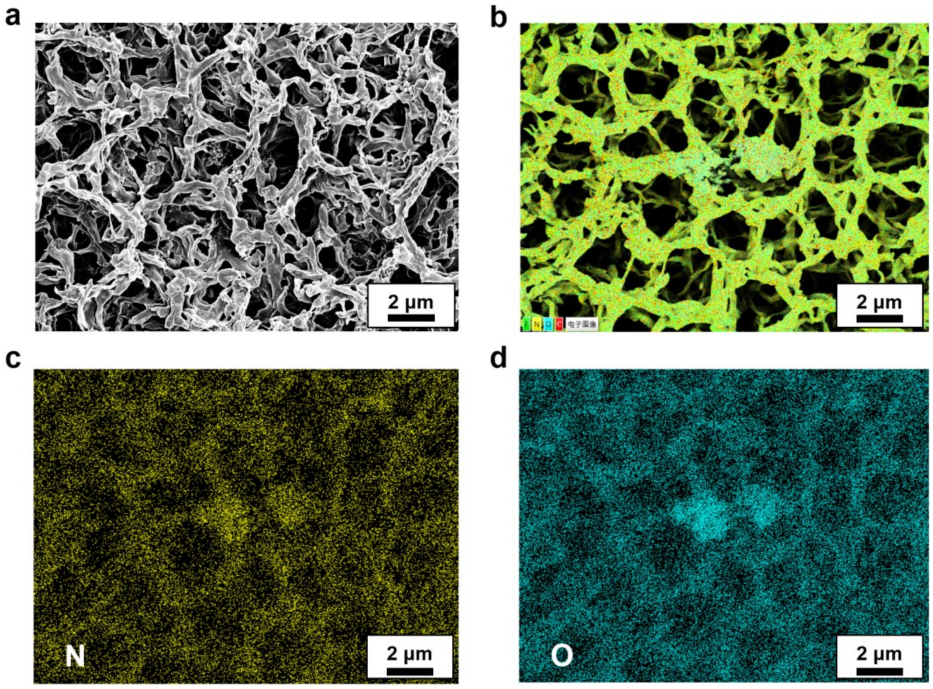

Fig. S1 Surface morphology and elemental analysis of the PVDF/PDA membrane. (a) Surface micrograph demonstrating the 1 μm scale pore on the PVDF/PDA membrane. (b) An overlay of the elemental map for the membrane demonstrates the distribution of fluoride (green), nitrogen (yellow), oxygen (cyan) and carbon (red) elements on the surface. (c) The isolated nitrogen distribution on the PVDF/PDA membrane. (d) The oxygen distribution on PVDF/PDA surface.

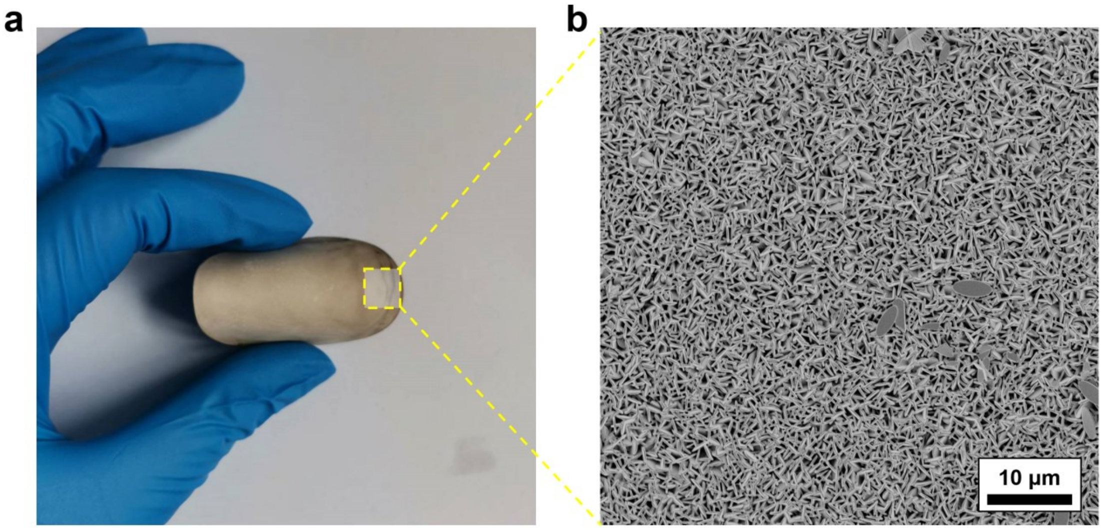

Fig. S2 (a) The photograph of a PVDF/PDA/ZIF-L membrane, demonstrates its robustness and is easily handled without significant fracturing. (b) An SEM micrograph taken after repeated bending and tearing, shows that the crystals remain attached to the surface without collapsing or peeling apart.

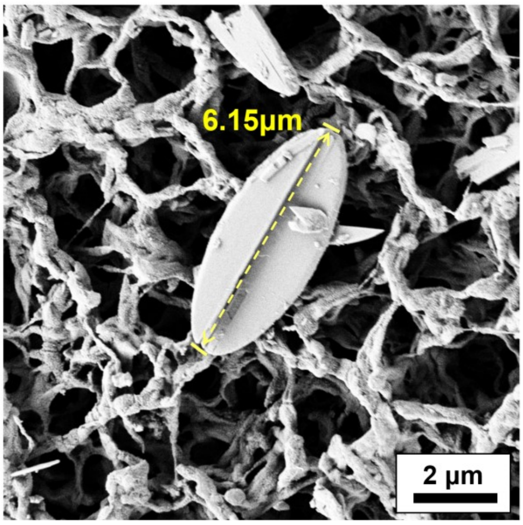

Fig. S3 SEM micrograph demonstrates a ZIF-L crystal that has been deposited, instead of grown on a PVDF surface without PDA modification.

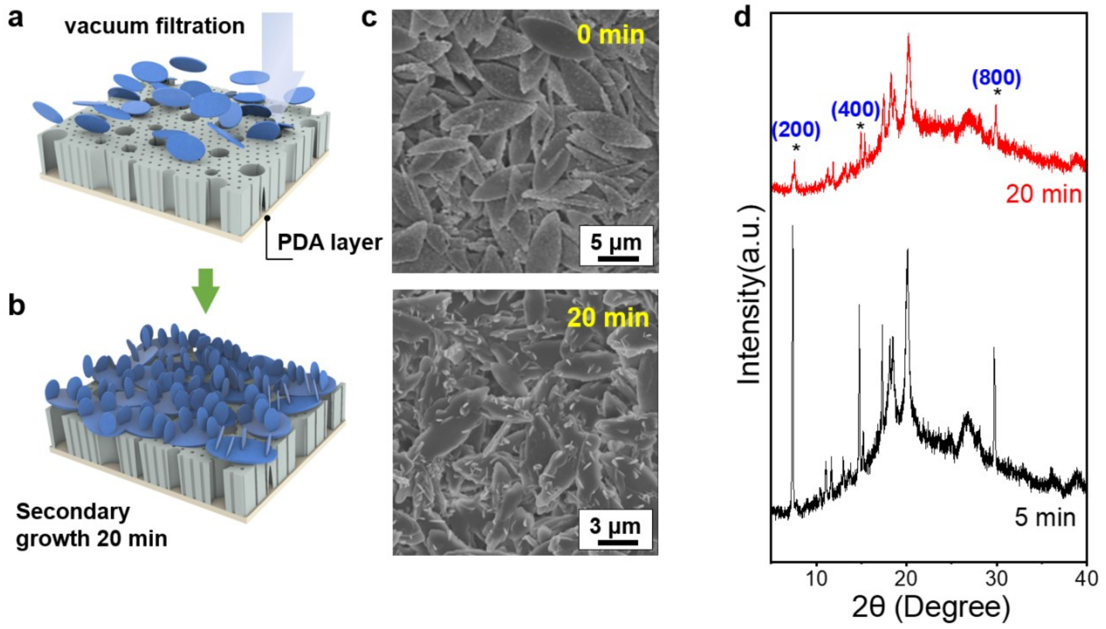

Fig. S4 (a) The in-plane alignment of parallel ZIF-L crystals with the support, achieved by vacuum filtration. The membrane was allowed for another 20 min secondary growth, which results in the formation of vertical ZIF-L crystals on the parallel surface shown in (b), with micrographs displayed in (c). (d) The XRD patterns of parallel ZIF-L membranes after 5 min and 20 min of secondary growth.

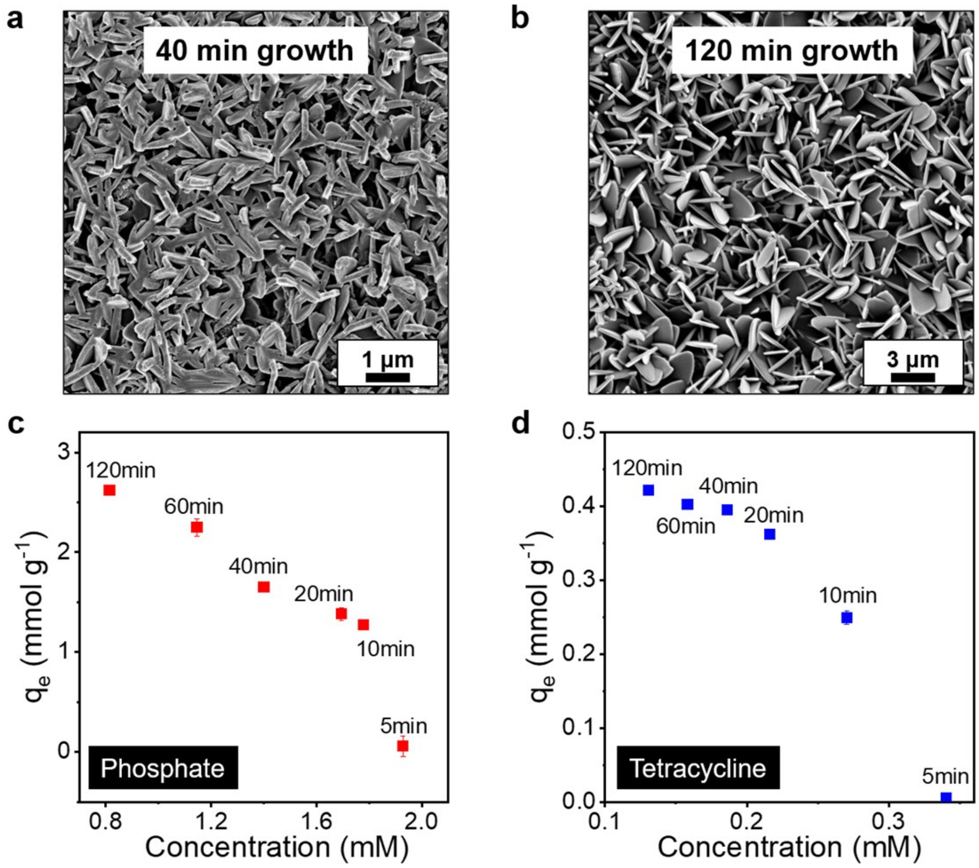

Fig. S5 SEM micrographs showcase the surface evolution of the PVDF/PDA/ZIF-L membrane as a function of growth time from (a) 40 min to (b) 120 min. Crystals aggregate closer to each one another with longer growth time. The equilibrium capacity of the ZIF-L membrane as a function of retentate concentration is listed with respect to (c) phosphate and (d) tetracycline over a specific growth time ranging from 5 to 120 min. A consistent ratio of 8:1 (Hmim: Zn^{2+}) is selected for this growth time adsorption experiment.

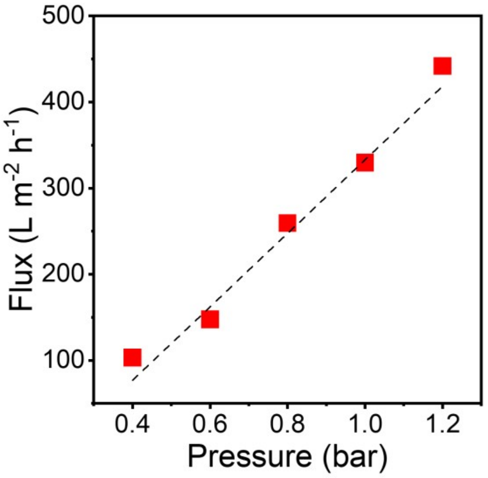

Fig. S6 Pure water flux of perpendicular oriented PVDF/PDA/ZIF-L as a function of trans-membrane pressure is presented. This analysis determines a hydraulic permeance of ~ 430 L m^{-2} h^{-1} bar^{-1}.

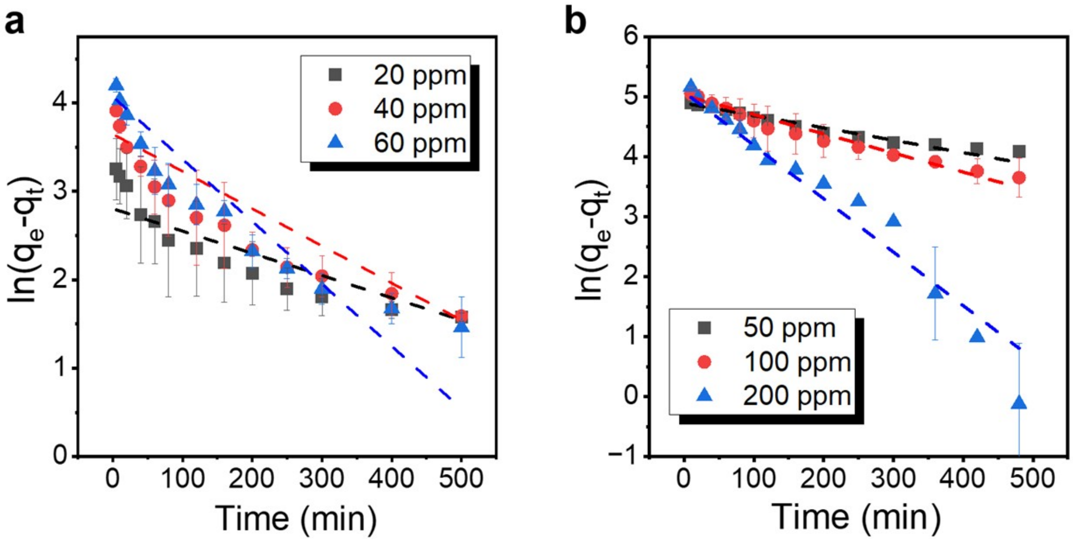

Fig. S7 Fittings of the pseudo-first order adsorption model to the static adsorption capacity of (a) total phosphorus and (b) tetracycline on the PVDF/PDA/ZIF-L membrane accounting capacities in mg g^{-1}.

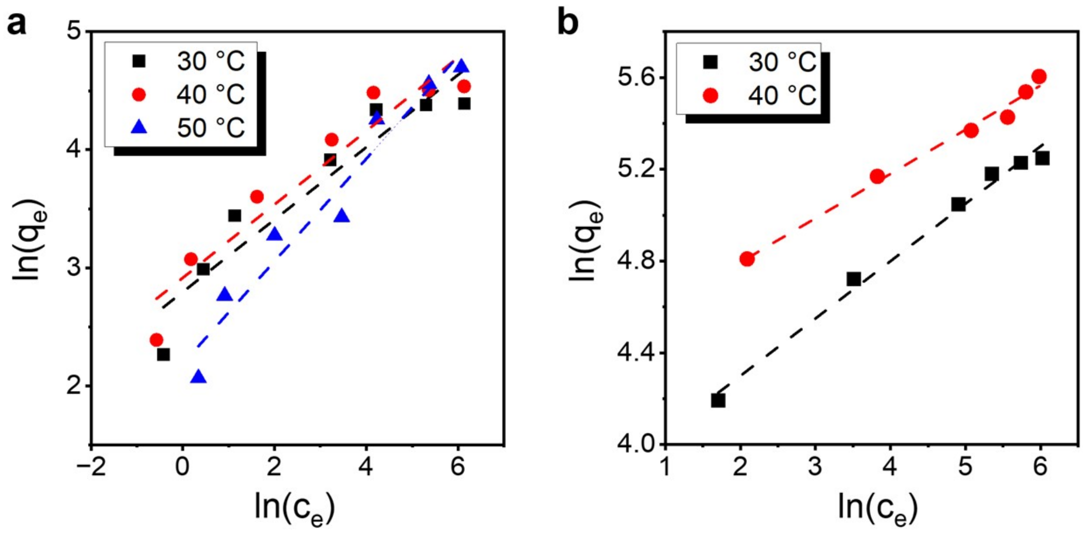

Fig. S8 Fittings of the Freundlich isotherm model to the static adsorption capacity of (a) total phosphorus and (b) tetracycline on the PVDF/PDA/ZIF-L membranes with a range of temperatures from 30, to 40 and 50 °C, where c_e is the retentate concentration in mg L^{-1} and q_e is the equilibrium capacity in mg g^{-1}.

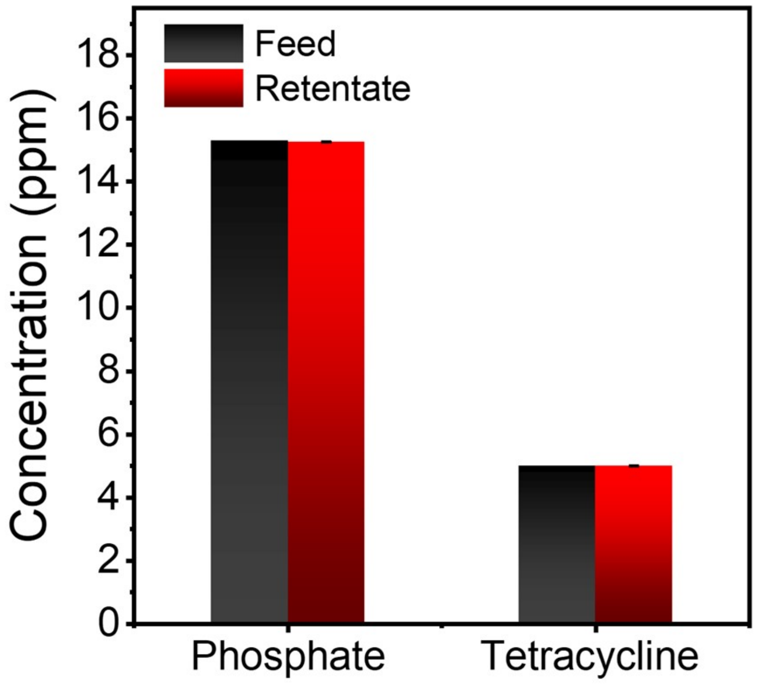

Fig. S9 The concentration of phosphate and tetracycline in the solution both before and after immersing a piece of PVDF/PDA membrane for a pre-determined duration for adsorption. The solution concentration remains consistent, indicating that there is no

significant adsorption.

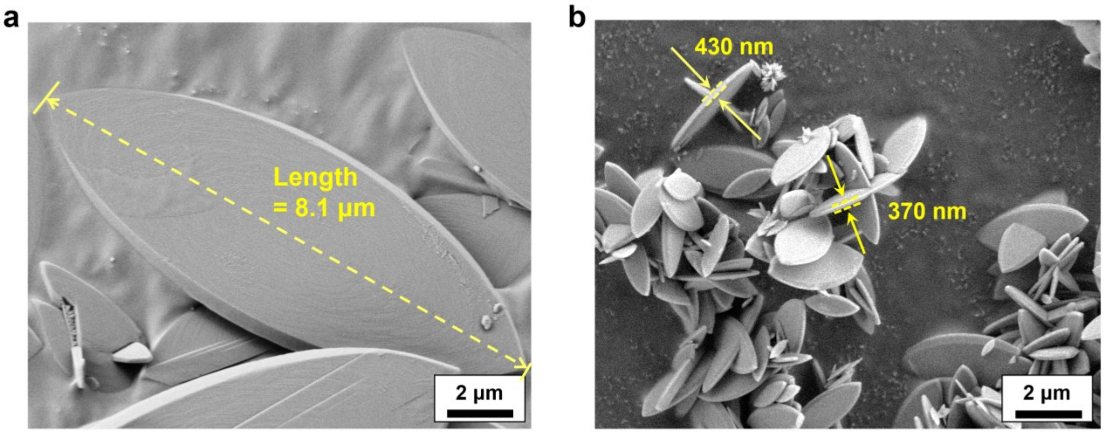

Fig. S10 Illustration for (a) the in-plane dimension and (b) the thickness of ZIF-L crystals fabricated by solution-based preparation protocols.

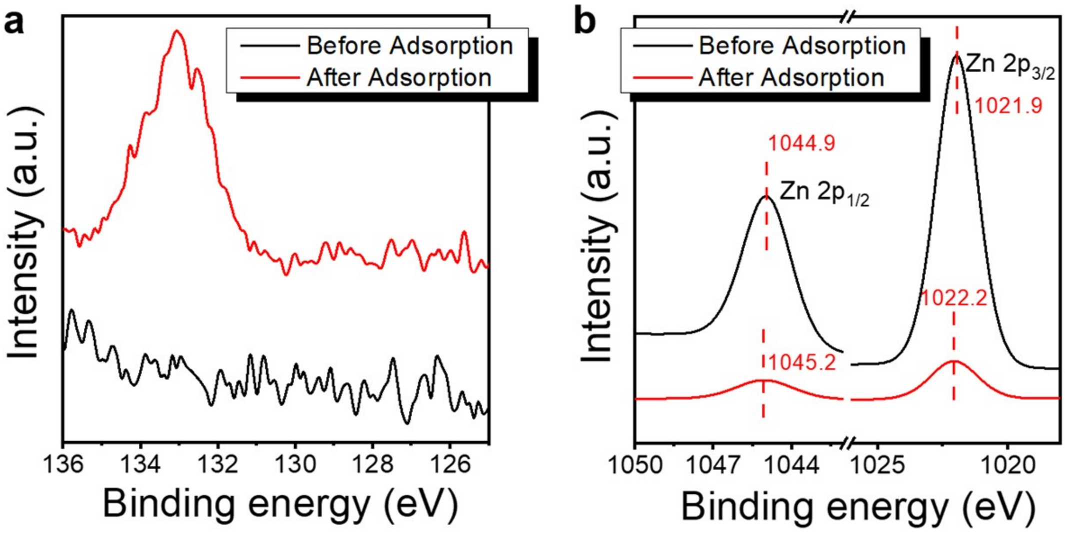

Fig. S11 XPS analysis of PVDF/PDA/ZIF-L membrane before and after phosphate adsorption on (a) P 2p and (b) Zn 2p spectra.

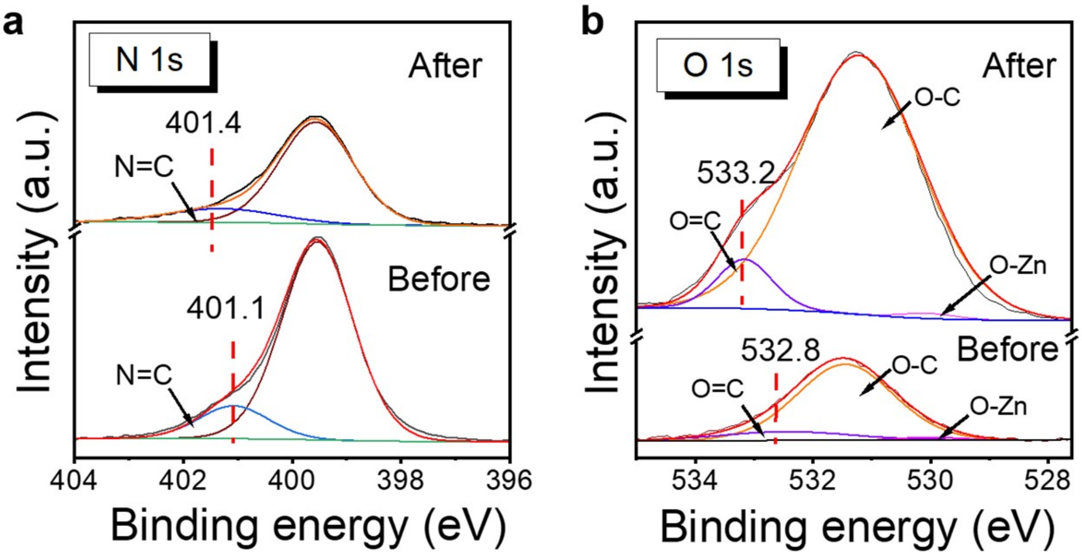

Fig. S12 XPS analysis of PVDF/PDA/ZIF-L membrane showing (a) N 1s and (b) O1s spectra before and after tetracycline adsorption.

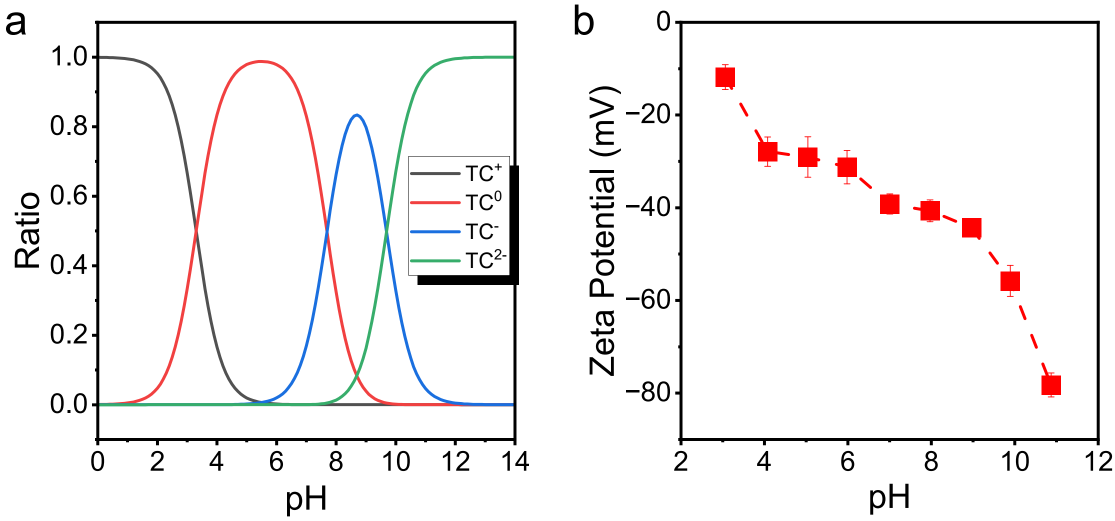

Fig. S13 (a) Calculated relative abundance of charged tetracycline species, in positive, neutral, and negative valences, as a function of solution pH. (b) Zeta potential of the PVDF/PDA/ZIF-L membrane measured against solution pH.

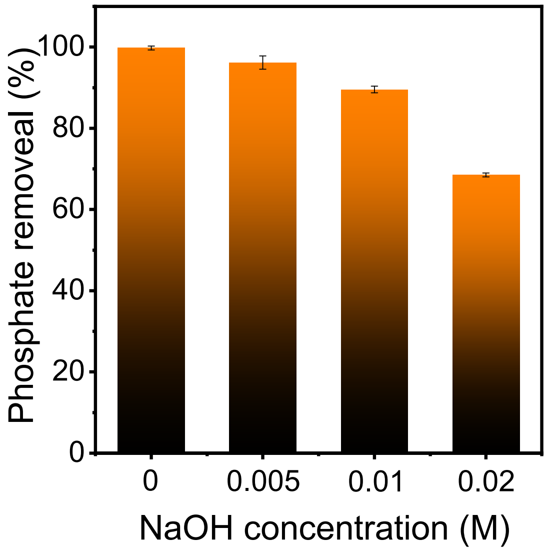

Fig. S14 The 15 ppm phosphate removal efficiency of the ZIF-L membranes, each regenerated by using different concentrations of NaOH in ethanol regeneration solution for 1 h.

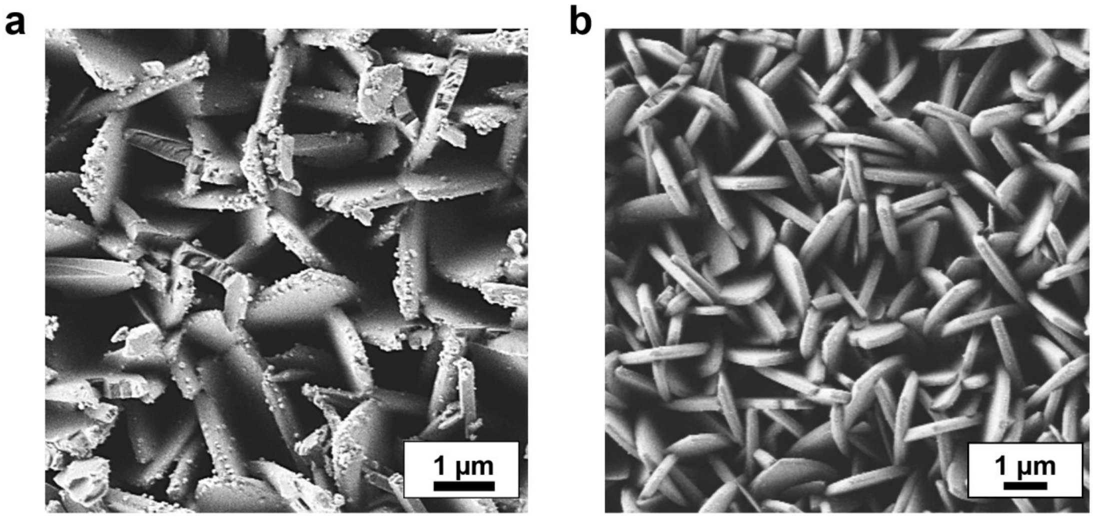

Fig. S15 (a) Surface SEM micrograph of the PVDF/PDA/ZIF-L membrane after being regenerated for 3 cycles using a 0.01 M NaOH ethanol solution. The crystals primarily remain intact, however, their surface is covered with powder-like precipitates. (b) membrane surface remains intact after being immersed in ethanol regeneration solution for 24 h.

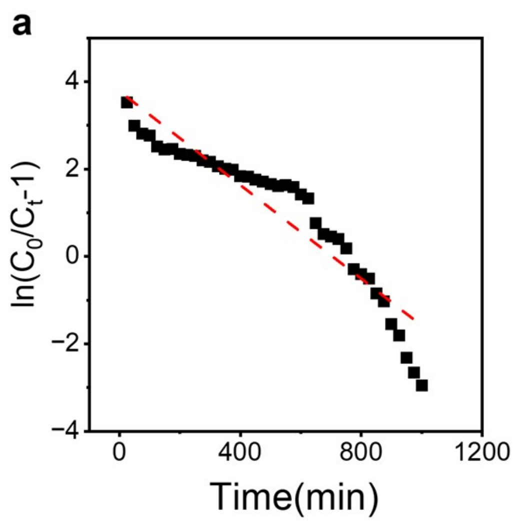

Fig. S16 Application of the Thomas model (red dashed line), incorporating the Langmuir isotherm and second-order adsorption kinetics, is applied to fit the breakthrough curves of (a) 10 ppm tetracycline and (b) 2 ppm total phosphorus (marked with black squares), using the composite PVDF/PDA/ZIF-L membranes. This model assumes zero axial dispersion.

3. Supplementary Tables

Table S1 Calculated kinetic parameters of phosphate adsorption by ZIF-L membranes at varying concentrations accounting total phosphorus concentration.

**Concentration of total phosphorus (mg L^{-1})**		**20**	**40**	**60**
**Pseudo First-order**	**K_1 (min^{-1})**	2.5×10^{-3}	4.2×10^{-3}	7.1×10^{-3}
	**q_e (mg g^{-1})**	16.5	38.4	58.6
	**R^2**	87.0%	96.0%	95.0%
**Pseudo second-order**	**K_2 (g mg^{-1} min^{-1})**	1.1×10^{-3}	6.4×10^{-4}	5.5×10^{-4}
	**q_e (mg g^{-1})**	27.3	53.0	75.8
	**R^2**	99.9%	99.6%	99.3%

Table S2 Calculated isotherm parameters of phosphate adsorption by ZIF-L

membranes at different temperatures accounting total phosphorus concentration.

Temperature (°C)	Langmuir			Freundlich		
	Q_m, total phosphorus (mg g^{-1})	K_L (L mg^{-1})	R^2	1/n (g mg^{-1} min^{-1})	K_F (L mg^{-1})	R^2
30	80.8	0.16	99.9%	0.31	16	89.5%
40	94.6	0.13	99.9%	0.31	19	91.5%
50	115	3.4×10^{-2}	99.6%	0.43	8.9	94.9%

Table S3 Phosphate adsorption capacity of inorganic sorbents recorded in literature accounting total phosphorus concentration.

Adsorbent	Packing ratio	K_L (L mg^{-1})	Q_m, total phosphorus (mg g^{-1})	Ref.
ZIF-8	0.5 g L^{-1} (25 °C)	0.94	38	[2]
ZIF-8	0.1 g L^{-1} (30 °C)	N/A	29	[3]
ZIF-8	0.2g L^{-1} (25 °C)	N/A	18	[4]
ZIF-L	0.2g L^{-1} (25 °C)	N/A	25	[4]
PVDF/PDA/ZIF-L	0.4 g L^{-1} (30 °C)	0.16	81	This work
PVDF/PDA/ZIF-L	0.4 g L^{-1} (40 °C)	0.13	95	This work
PVDF/PDA/ZIF-L	0.4 g L^{-1} (50 °C)	0.034	115	This work
Magnesium hydroxide	0.6 g L^{-1} (25 °C)	0.31	5.2	[5]

Calcinated Zn-Al LDH	N/A (25 °C)	4.0	19	[6]
Hydrous cerium oxide	2 g L^{-1} (25 °C)	0.84	33	[7]
Zirconium oxide	0.2 g L^{-1} (25 °C)	0.39	22	[8]

Table S4 Calculated kinetic parameters of tetracycline adsorption by ZIF-L membranes at different concentrations.

**Concentration (mg L^{-1})**	**Pseudo first order**			**Pseudo second order**		
	**k_1 (min^{-1})**	**q_e (mg g^{-1})**	**R^2**	**k_2 (g mg^{-1} min^{-1})**	**q_e (mg g^{-1})**	**R^2**
**50**	2.1×10^{-3}	134	97.1%	3.47×10^{-5}	139	98.3%
**100**	3.2×10^{-3}	152	98.5%	4.08×10^{-5}	179	99.7%
**200**	8.9×10^{-3}	161	95.8%	1.11×10^{-4}	222	98.9%

Table S5 Calculated isotherms parameters of tetracycline adsorption by ZIF-L membranes at various temperatures.

**Temperature (°C)**	**Langmuir**			**Freundlich**		
	**Q_m (mg g^{-1})**	**K_L (L mg^{-1})**	**R^2**	**1/n (g mg^{-1} min^{-1})**	**K_F (L mg^{-1})**	**R^2**
**30**	196	5.0×10^{-2}	99.8%	0.25	45	99.0%
**40**	294	3.0×10^{-2}	98.6%	0.19	82	97.7%

Table S6 Tetracycline adsorption capacity of other inorganic adsorbents recorded in literature.

**Adsorbent**	**Packing ratio**	**K_L (L mg^{-1})**	**Q_m (mg·g^{-1})**	**Ref.**
Fe_3O_4@PDA-ZIF-8	0.13 g L^{-1}	5.4×10^{-3}	111	[9]
PVDF/PDA/ZIF-L	0.40 g L^{-1} (30	0.050	196	This work

	°C)			
PVDF/PDA/ZIF-L	0.40 g L^{-1} (40 °C)	0.030	294	This work
Magnetic functionalized biochar	0.25 g L^{-1} (35 °C)	0.095	115	[10]
Fe_3O_4@PDA@Eu-MOF	0.20 g L^{-1}	0.093	145	[11]
Carbon-doped boron nitride (BCN)	0.40 g L^{-1}	0.27	120	[12]

References

1. X. Zhang, H. Li, W. Miao, Q. Shen, J. Wang, D. Peng, J. Liu and Y. Zhang, *AIChE Journal*, 2019, **65**, e16596.
2. M. Shams, M. H. Dehghani, R. Nabizadeh, A. Mesdaghinia, M. Alimohammadi and A. A. Najafpoor, *J.Mol.Liq.*, 2016, **224**, 151-157.
3. Y. Wang, W. Zhao, Z. Qi, L. Zhang, Y. Zhang, H. Huang and Y. Peng, *Chem. Eng.J.*, 2020, **394**, 124992.
4. C. Huang, H. Zhang, K. Zheng, Z. Zhang, Q. Jiang and J. Li, *Sci.Total. Environ.*, 2021, **785**, 147382.
5. J. Lin, S. He, X. Wang, H. Zhang and Y. Zhan, *Colloid. Surface. A*, 2019, **561**, 301-314.
6. X. Cheng, X. Huang, X. Wang, B. Zhao, A. Chen and D. Sun, *J. Hazard. Mater.*, 2009, **169**, 958-964.
7. H. Guo, W. Li, H. Wang, J. Zhang, Y. Liu and Y. Zhou, *Rare Metals*, 2011, **30**, 58-62.
8. J. Lin, X. Wang and Y. Zhan, *J.Environ.Sci.*, 2019, **76**, 167-187.
9. D. Sheng, X. Ying, R. Li, S. Cheng, C. Zhang, W. Dong and X. Pan, *Chemosphere*, 2022, **308**, 136249.

10. F. Zhang, J. Wang, Y. Tian, C. Liu, S. Zhang, L. Cao, Y. Zhou and S. Zhang, *Environ. Pollut.*, 2023, 121681.
11. J. Li, R. Yao, B. Deng, Z. Li, K. Tuo, C. Fan, G. Liu and S. Pu, *Chem. Eng.J.*, 2023, **464**, 142626.
12. Y. Guo, C. Yan, P. Wang, L. Rao and C. Wang, *Chem. Eng.J.*, 2020, **387**, 124136.
